# Two‐Dimensional Flash Memory: Materials, Interfaces, and Storage–Computing Convergence

**DOI:** 10.1002/advs.78009

**Published:** 2026-09-30

**Authors:** Zhiyuan Wang, Fanyi Meng, Fanying Meng, Shida Huo, Junxi Xu, Yaohui Wang, Yuexuan Ma, Yuan Xie, Xiaodong Hu, Caofeng Pan, Enxiu Wu

**Affiliations:** ^1^ State Key Laboratory of Precision Measurement Technology and Instruments School of Precision Instruments and Opto‐electronics Engineering Tianjin University Tianjin China; ^2^ School of Microelectronics Tianjin University Tianjin China; ^3^ School of Electronics and Information Engineering Tiangong University Tianjin China; ^4^ Beijing Key Laboratory for Atomic Manufacturing Equipment and Intelligent Sensing Institute of Atomic Manufacturing Beihang University Beijing China

**Keywords:** 2D materials, flash memory, interface engineering, in‐memory computing

## Abstract

Flash memory remains the dominant nonvolatile storage technology, yet its continued scaling is increasingly constrained by the coupled requirements of fast programming, low operating energy, and long‐term reliability. Recent advances in 2D materials and van der Waals heterostructures provide an opportunity not merely to miniaturize flash cells, but to reconfigure the charge‐programming physics itself. In this Review, we introduce interface‐controlled charge programming as a unifying framework for understanding and designing 2D flash memories. We discuss how atomically thin channels, clean van der Waals interfaces, engineered tunneling barriers, controllable trap states, and contact modulation regulate charge injection, transport, trapping, and confinement. This framework connects device‐level metrics, including nanosecond‐to‐subnanosecond programming, low‐energy operation, long retention, and high endurance, with emerging system‐level functions such as multilevel storage, in‐memory computing, sensing‐memory‐computing integration, and CMOS‐compatible heterogeneous integration. We further identify critical challenges in wafer‐scale synthesis, device variability, reliability benchmarking, and array‐level implementation. By shifting flash memory design from bulk‐field‐driven tunneling to interface‐programmed charge control, 2D materials may enable a new class of nonvolatile devices for storage–computing convergence beyond conventional silicon flash.

## Graphical abstract

We systematically summarize recent advances in 2D flash memory, highlighting how interface and material engineering can overcome the conventional trade‐offs among speed, power consumption, and reliability in silicon‐based flash, and outlining its potential for high‐performance memory and in‐memory computing applications.

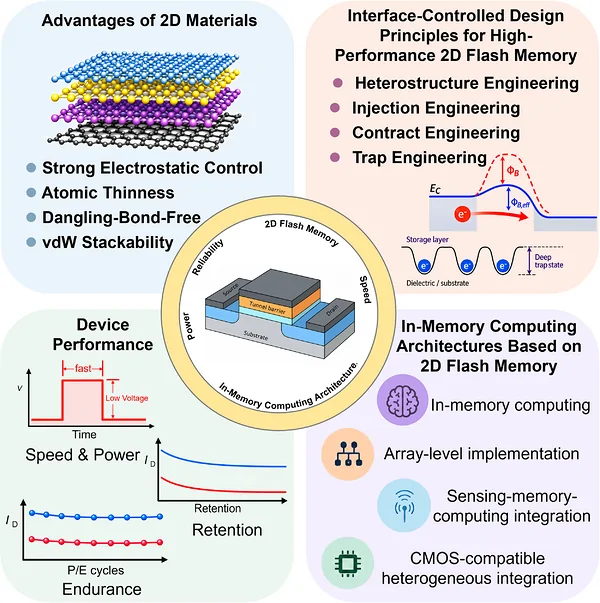

## 1 Introduction

The rapid growth of data generated by artificial intelligence, the Internet of Things, edge devices, and data‐intensive computing is placing increasingly stringent demands on memory hardware, including higher storage density, faster operating speed, lower energy consumption, improved cycling endurance, and longer data retention [1, 2, 3, 4, 5]. Among existing memory technologies, flash memory remains the dominant nonvolatile storage platform because of its high integration density, low cost, mature manufacturing infrastructure, and robust data‐retention capability. It therefore continues to serve as a cornerstone of modern information systems, from embedded electronics to large‐scale data storage.

Despite its technological maturity, conventional silicon‐based flash memory is approaching the physical limits of continued scaling. As device dimensions are reduced toward the sub‐10 nm regime, planar flash cells suffer from aggravated short‐channel effects, increased leakage current, enhanced cell‐to‐cell interference, and degraded dielectric reliability [6, 7, 8, 9]. These scaling‐induced issues not only restrict further improvements in storage density, but also intensify the long‐standing trade‐offs among programming speed, operating voltage, endurance, and data retention [10, 11, 12]. 3D NAND architectures have extended the lifetime of flash technology by replacing lateral scaling with vertical stacking; however, further increases in the number of stacked layers bring rapidly rising fabrication complexity, process cost, structural nonuniformity, and device variability, thereby posing critical challenges for manufacturability, yield, and long‐term reliability [13, 14, 15].

More fundamentally, these limitations are not merely consequences of device geometry, but originate from the charge‐programming physics of conventional flash memory. In silicon‐based flash devices, charge injection and removal are typically achieved through high‐field Fowler‐Nordheim tunneling or hot‐carrier injection across dielectric barriers [16]. Although these mechanisms enable nonvolatile charge storage, faster programming usually requires stronger electric fields, which inevitably increase energy dissipation, promote defect generation in the tunneling dielectric, and accelerate long‐term reliability degradation [17, 18, 19]. As a result, programming speed, power consumption, and reliability are intrinsically coupled, making simultaneous optimization of these key metrics difficult within existing silicon‐based material and device frameworks [20, 21, 22].

2D materials offer a distinct route to reconfigure this charge‐programming process. Representative 2D materials, including graphene, transition‐metal dichalcogenides (TMDs), and hexagonal boron nitride (hBN), possess atomically thin bodies, dangling‐bond‐free surfaces, strong electrostatic gate control, and van der Waals integration capability [23, 24, 25]. These features can suppress short‐channel effects, reduce interface‐related disorder, and enable the construction of atomically sharp heterointerfaces without the strict lattice‐matching constraints of conventional epitaxy. More importantly, 2D materials provide new degrees of freedom for engineering charge injection pathways, tunneling barriers, storage layers, trap states, and interfacial band alignment [26, 27, 28, 29, 30, 31]. Here, “interface‐controlled charge programming” refers to a device‐design strategy centered on interface engineering. By tailoring the contact, tunneling and charge‐storage interfaces, together with their relative band alignments, carrier injection, transfer and retention can be jointly regulated to optimize programming efficiency and operational stability. Therefore, charge programming in 2D flash memory can evolve from conventional bulk‐field‐driven tunneling toward interface‐controlled processes governed by band‐structure design and interfacial coupling.

In this context, the significance of 2D materials extends beyond further miniaturization of flash memory cells. They provide an opportunity to reconstruct the physical basis of flash operation by controlling the full lifecycle of stored charges, including injection, transport, trapping, and confinement. Through rational material selection and van der Waals heterostructure engineering, the injection barrier, tunneling distance, trap depth, leakage pathway, and local electric‐field distribution can be modulated with high precision. This interface‐controlled charge‐programming framework offers a potential route to alleviate the conventional trade‐offs among speed, power consumption, retention, and endurance, while also enabling functional integration with in‐memory computing, sensing, and heterogeneous CMOS‐compatible systems [32, 33].

In this Review, we discuss recent advances in 2D flash memory from the perspective of interface‐controlled charge programming. We first revisit the evolution of conventional flash memory and clarify the physical bottlenecks associated with scaling, high‐field programming, and dielectric reliability. We then summarize the unique advantages of 2D materials and van der Waals heterostructures for flash memory, followed by a critical analysis of representative strategies for achieving ultrafast operation, low‐power programming, long data retention, and high endurance. Finally, we highlight emerging developments in array‐level implementation, in‐memory computing, sensing‐memory‐computing integration, and CMOS‐compatible heterogeneous integration, and discuss the remaining challenges in manufacturing, device uniformity, reliability benchmarking, and system‐level deployment. The overall framework of this Review is summarized in Figure 1.

**FIGURE 1:**
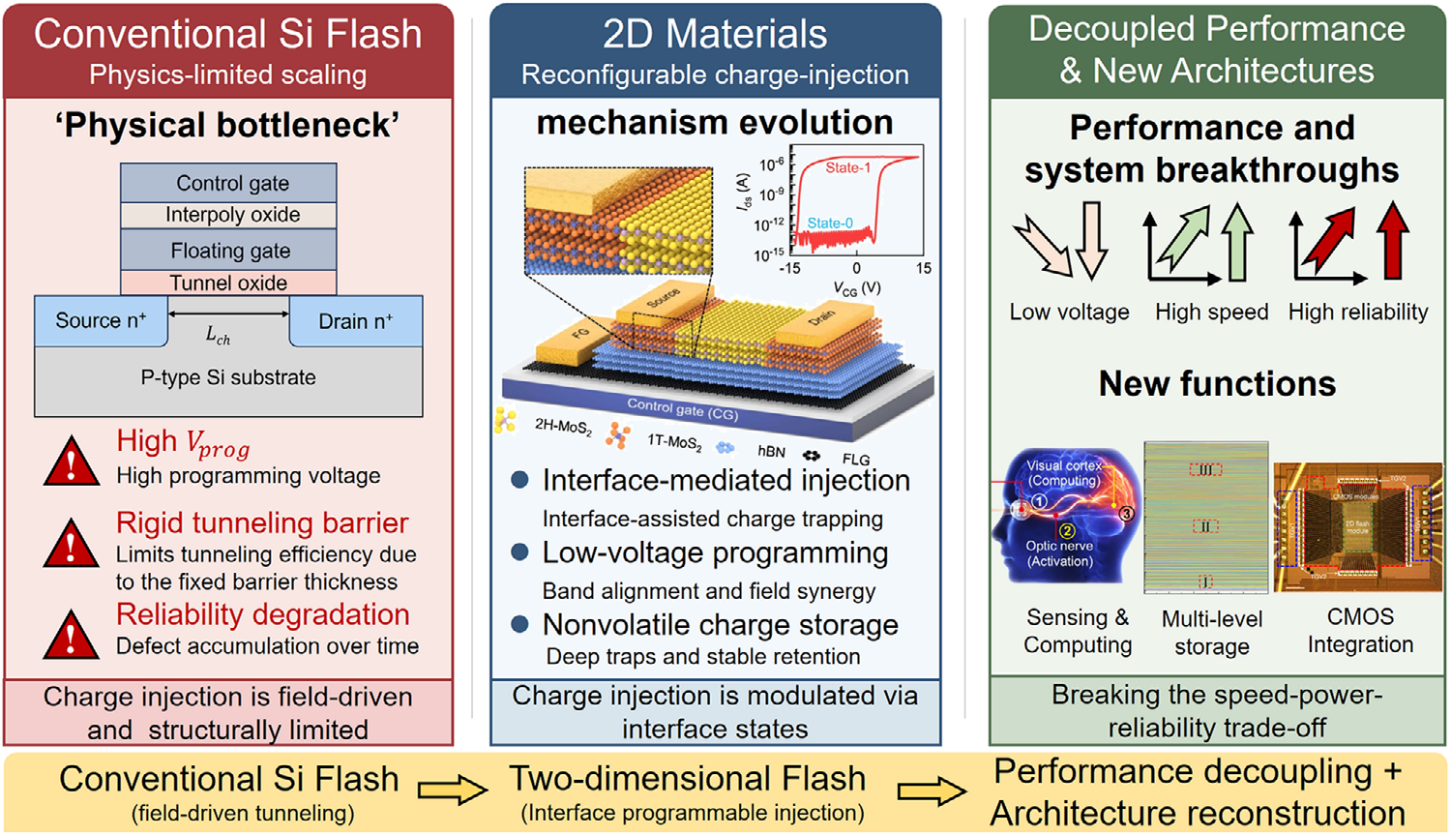
Overall framework of this Review [34, 35, 36, 37]. Reproduced under terms of the CC BY 4.0 license [35]. Copyright 2023, Springer Nature. Reproduced with permission [35]. Copyright 2025, Springer Nature. Reproduced under terms of the CC BY 4.0 license [36]. Copyright 2026, Springer Nature. Reproduced with permission [37]. Copyright 2025, Springer Nature. The Review traces the evolution of flash memory from conventional silicon‐based architectures to 2D‐material‐based devices, highlights interface‐controlled charge programming as a unifying design principle, summarizes representative strategies for optimizing speed, power consumption, retention, and endurance, and outlines emerging applications of 2D flash memory in in‐memory computing, array integration, sensing‐memory‐computing systems, and CMOS‐compatible heterogeneous platforms.

Unlike previous reviews that primarily classify 2D memory devices by material type or device architecture, this Review uses charge programming as the central organizing principle. We focus on how interfaces determine the full charge lifecycle—carrier injection, interfacial transport, trap occupation, charge confinement, and charge release—and how these processes collectively define the speed‐power‐reliability envelope of 2D flash memories. This perspective allows recent progress in ultrafast operation, low‐power programming, retention enhancement, endurance improvement, and storage‐computing integration to be discussed within a single physical framework.

## 2 Development of Flash Memory Technology

### 2.1 Evolution of Flash Memory: From Floating‐Gate Concept to 3D Integration and Beyond

The development of flash memory has progressed through a series of paradigm shifts driven by advances in charge‐storage mechanisms, device architectures, and integration strategies (Figure 2). Flash memory originated from the floating‐gate MOSFET proposed by Dawon Kahng and Simon Sze in 1967 [38], in which a conductive floating gate is embedded between the control gate and the channel and fully isolated by insulating layers. Charges stored in the floating gate modulate the threshold voltage of the transistor, thereby enabling nonvolatile information storage through electrical programming.

**FIGURE 2:**
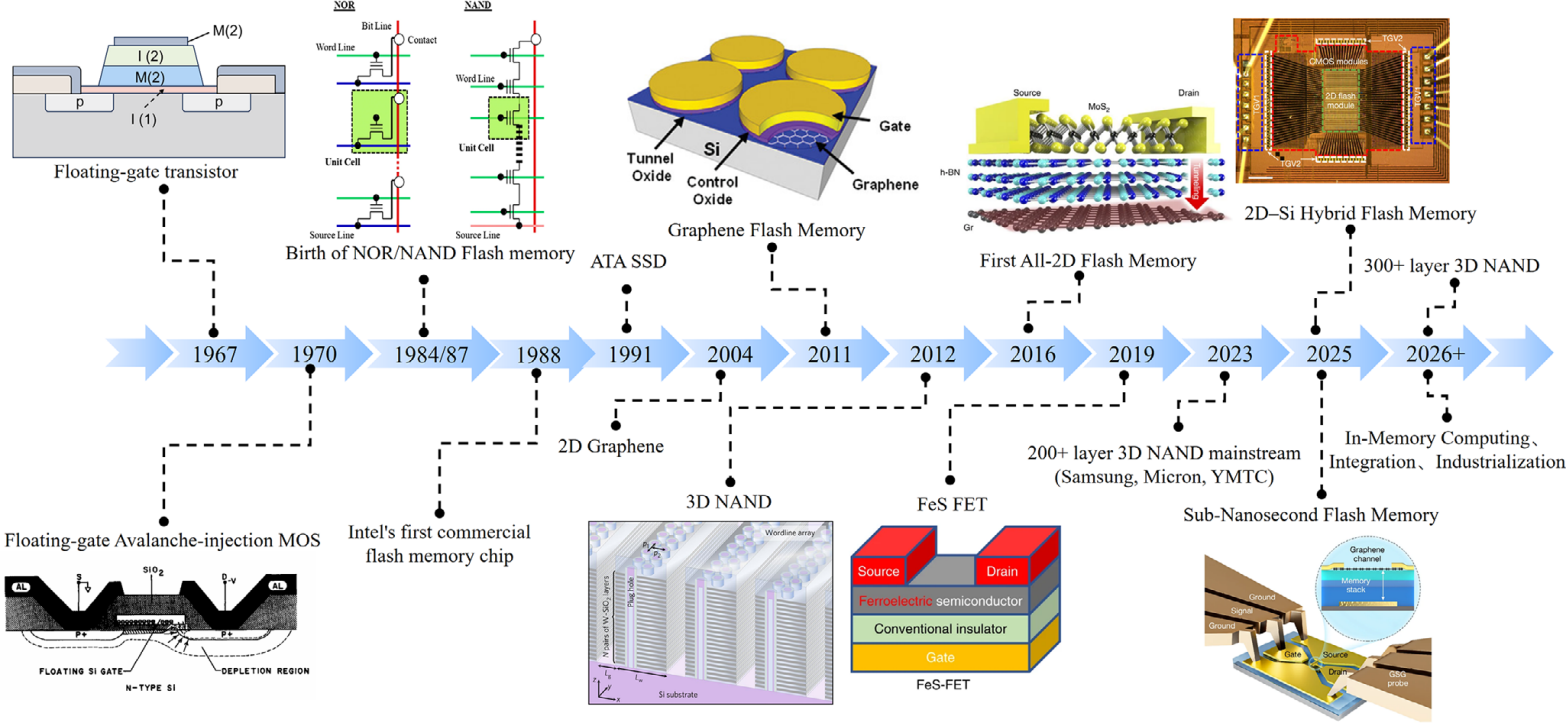
Historical evolution and basic device concepts of flash memory [37, 39, 40, 41, 42, 43, 44, 45]. Reproduced with permission [37]. Copyright 2025, Springer Nature. Reproduced with permission [39]. Copyright 2011, American Chemical Society. Reproduced under terms of the CC BY 4.0 license [40]. Copyright 2016, Springer Nature. Reproduced with permission [41]. Copyright 2019, Springer Nature. Reproduced with permission [42]. Copyright 1971, AIP Publishing. Reproduced under terms of the CC BY 4.0 license [43]. Copyright 2014, Springer Nature. Reproduced with permission [44]. Copyright 2018, Springer Nature. Reproduced with permission [45]. Copyright 2025, Springer Nature.Timeline showing the development of flash memory from the floating‐gate concept and EPROM to NAND flash, 3D NAND, and emerging 2D‐material‐based flash technologies.

Building on this concept, Dov Frohman developed EPROM in 1970 [46], which was subsequently commercialized by Intel in 1971, marking the first practical realization of floating‐gate memory. Although EPROM demonstrated nonvolatile operation, its reliance on ultraviolet erasure limited its practical applicability. A decisive breakthrough occurred in 1984 [47], when Fujio Masuoka introduced the block‐erase concept, enabling the simultaneous erasure of large memory regions and establishing the fundamental operational principle of flash memory. Subsequent commercialization by Intel, together with SanDisk's efforts to position flash as a universal data‐storage medium, transformed flash from a code‐storage technology into a mainstream storage platform.

During the 1990s and early 2000s, the central challenge in flash memory development shifted to density scaling and cost reduction. NAND flash gradually replaced NOR flash as the dominant architecture due to its series‐connected cell configuration, which offers significantly higher storage density and lower cost per bit. This transition was further accelerated by the introduction of multi‐level cell (MLC) technology, enabling multiple charge states to be stored within a single memory cell and thereby substantially improving storage efficiency.

However, as planar NAND flash scaled into the nanometer regime, device dimensions approached fundamental physical limits. Increasing cell‐to‐cell interference, increased charge leakage, and degraded reliability constrained further lateral scaling. This triggered a second major paradigm shift—from planar miniaturization to vertical integration—leading to the emergence of 3D NAND [48]. Since the introduction of 32‐layer V‐NAND in 2012, the number of vertically stacked layers has increased to more than 200, significantly enhancing storage density and throughput [49, 50].

Despite its industrial success, 3D NAND does not fundamentally alter the underlying charge programming physics of conventional flash memory. Its operation still relies on charge transfer through insulating barriers and remains constrained by tunnel dielectric reliability, programming speed, and energy consumption. Consequently, the future development of flash memory is increasingly shifting from purely structural scaling toward materials‐ and interface‐driven innovation.

In this context, 2D materials have emerged as promising candidates for next‐generation flash technologies. Since the discovery of graphene in 2004 [51], 2D materials have attracted widespread attention owing to their atomic thickness, high carrier mobility, and dangling‐bond‐free van der Waals interfaces. Subsequent demonstrations of graphene‐based flash concepts [39], all‐2D flash devices [40], ferroelectric semiconductor field‐effect transistors [41], and hybrid 2D‐silicon flash systems [37] suggest that flash memory is entering a new stage in which material selection and interface engineering become as critical as device architecture [52].

Looking forward, while industry continues to push 3D NAND toward stacking levels exceeding 300 layers [53], emerging flash technologies based on novel materials are beginning to exhibit switching performance in selected metrics approaching that of volatile memories. More importantly, flash memory is evolving from a passive storage medium into an active information‐processing platform, with increasing potential for logic operations, neuromorphic functionality, and data‐centric computing [54, 55, 56].

### 2.2 Flash Architectures: Array Organization and Storage Mechanisms

Flash memory architectures are primarily categorized into NOR and NAND configurations (Figure 3), which differ intrinsically in cell connectivity and access mode [57].

**FIGURE 3:**
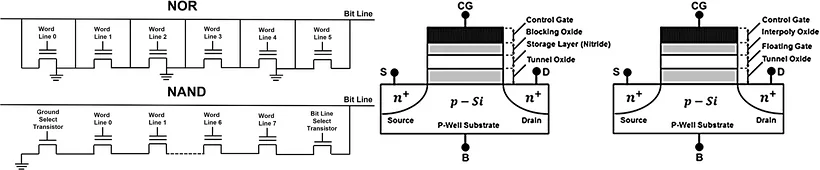
Fundamental operating principles of conventional silicon‐based flash memory [58]. Reproduced with permission [58]. Copyright 2016, Springer Nature. Schematic illustration of charge injection, charge storage, and threshold‐voltage modulation in nonvolatile flash operation. Charges injected into the floating gate or charge‐trap layer modulate the channel conductance and define different memory states, while charge removal resets the device to the erased state [59, 60].

NOR flash adopts a parallel architecture in which each memory cell is directly connected to both the bit line and the word line, enabling true random access [61]. This architecture supports direct addressing and fast reading operations, with access times typically in tens of nanoseconds. Consequently, NOR flash is widely employed in applications requiring rapid code execution, such as boot memory in embedded systems, mobile devices, industrial controllers, and automotive electronics. In addition, NOR flash offers excellent byte‐level programmability and strong disturbance immunity, making it well suited for low‐density but high‐reliability storage scenarios [61].

In contrast, NAND flash employs a series‐connected string architecture in which multiple memory cells are linked in series and share common bit lines. This configuration reduces interconnect complexity, improves area efficiency, and significantly increases storage density while lowering cost per bit. Although NAND flash exhibits slower random‐access performance—since an entire string must be activated before accessing an individual cell [62, 63]—it provides superior sequential read/write performance and scalability, making it the dominant technology for large‐capacity storage [64]. Its block‐level erase scheme further aligns well with high‐throughput data storage requirements [65].

At the cell level, modern flash memories mainly rely on two storage concepts: floating‐gate (FG) and charge‐trap‐layer (CTL) structures (Figure 3). In FG devices, charges are stored in a continuous conductive node—typically polysilicon—embedded within insulating layers. Charge injection and removal are typically achieved through Fowler‐Nordheim tunneling, and the stored charge modulates the threshold voltage to represent different memory states. With their mature fabrication processes and excellent retention characteristics, FG structures dominated early flash memory development; however, their scalability is increasingly limited by capacitive coupling, dielectric reliability, and cell‐to‐cell interference [66, 67, 68].

By contrast, CTL devices store charges in localized trap states within insulating materials rather than in a continuous conductive node. This discrete storage mechanism reduces sensitivity to local defects and leakage, as the failure of individual traps has a limited impact on the overall storage state [69, 70]. Furthermore, the insulating nature of the trapping layer suppresses parasitic coupling between adjacent cells, making CTL structures more compatible with aggressive scaling and 3D integration [71, 72]. As a result, CTL architectures have become the dominant storage mechanism in advanced NAND technologies.

### 2.3 Unique Advantages of 2D Materials for Flash Memory

Conventional flash memory is based on silicon technology. Although planar and 3D silicon‐based flash architectures have sustained the development of high‐density storage for decades, continued scaling has pushed these systems increasingly close to intrinsic physical limits [73]. As gate lengths shrink, short‐channel effects become more pronounced, leakage currents increase, and electrostatic control weakens, resulting in progressively degraded device performance [74].

The semiconductor industry has responded by transitioning from planar transistors to 3D architectures such as FinFETs, GAAFETs, and CFETs, along with materials innovations including strained silicon and high‐k/metal‐gate technologies [75, 76, 77, 78, 79, 80]. However, these advances do not eliminate the intrinsic material limitations of bulk silicon. As illustrated in Figure 4a, aggressive scaling induces pronounced short‐channel effects, leading to degraded electrostatic control and drain‐induced barrier lowering (DIBL). In particular, the presence of dangling bonds, interface roughness, and defect‐induced scattering further degrades carrier transport and gate control at deeply scaled dimensions.

**FIGURE 4:**
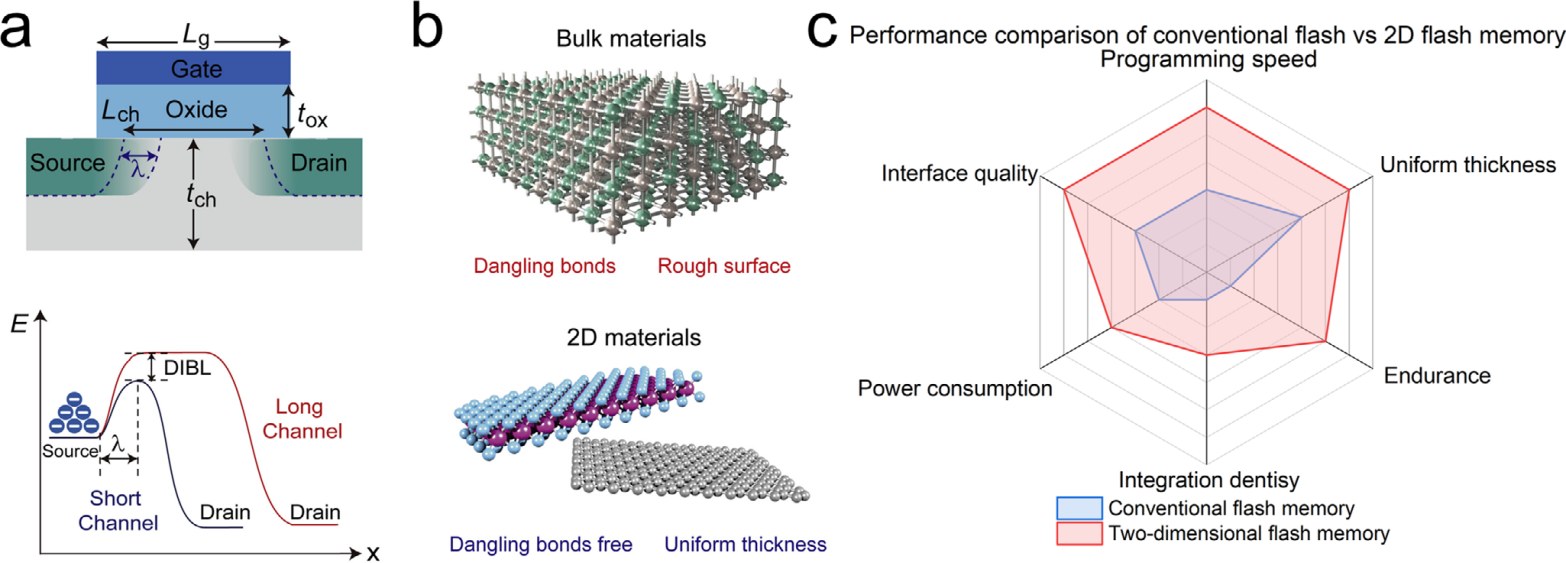
Physical scaling limitations of conventional silicon devices and the advantages of 2D materials for flash memory. (a) Schematic illustration of short‐channel effects and degraded electrostatic control in bulk semiconductor devices, including device structure and energy band diagrams showing DIBL [81]. Reproduced with permission [81]. Copyright 2022, Elsevier. (b) Comparison between bulk semiconductors and 2D layered materials, highlighting atomic thickness, dangling‐bond‐free surfaces, and enhanced gate control [81]. Reproduced with permission [81]. Copyright 2022, Elsevier. (c) Representative comparison between conventional flash memory and 2D flash memory in terms of programming speed, operating voltage, energy consumption, retention, endurance, and integration potential.

In contrast, 2D materials—including TMDs such as MoS_2_ and WS_2_—provide a different platform for flash memory. Figure 4b highlights that 2D materials, owing to their atomically thin channels, dangling‐bond‐free surfaces, and highly uniform layered structures enable superior electrostatic control and reduced interface scattering, making them particularly attractive for suppressing short‐channel effects and extending the scaling limits of memory devices.

Beyond electrostatic advantages, the most transformative feature of 2D materials lies in their compatibility with van der Waals heterostructures. These heterostructures enable atomically sharp, low‐defect interfaces, allowing precise engineering of tunneling barriers, charge trapping layers, and carrier injection pathways [82, 83, 84, 85]. As a result, 2D flash memory can achieve multidimensional performance enhancements compared with conventional silicon‐based flash, as summarized in Figure 4c.

Table 1 highlights that the advantages of 2D flash memory do not arise from a single performance metric, but from a fundamental shift in the underlying charge‐programming physics. In conventional silicon flash, charge injection is governed by high‐field‐driven tunneling processes, which intrinsically couple programming speed, power consumption, and reliability. In contrast, 2D flash memory leverages atomically sharp van der Waals interfaces and highly tunable band structures to enable interface‐controlled charge programming. This transition allows the injection pathway, barrier height, and trapping dynamics to be engineered independently, thereby partially decoupling the long‐standing trade‐off among speed, power, and endurance. As a result, 2D flash memory offers a unified platform for achieving ultrafast operation, low‐energy programming, and improved reliability, while also providing additional flexibility for multifunctional integration beyond conventional storage applications.

**TABLE 1:** Structural and performance comparison between conventional silicon‐based flash memory and 2D‐material‐based flash memory.

Metric	Conventional silicon flash	2D flash memory	Physical origin	Implication
Structure	Silicon MOS architecture	van der Waals heterostructures with configurable channel/tunneling/storage layers	Atomically sharp interfaces and flexible stacking	Breaks scaling limits and enables structure reconfigurability
Channel characteristics	Bulk Si channel with strong short‐channel effects and surface scattering	Atomically thin channels with strong electrostatic control	Reduced thickness and suppressed interface scattering	Improved gate control and scalability
Programming speed	µs–ms	ns to sub‐ns	Engineered injection pathways and reduced barriers	1‐3 orders of magnitude faster operation
Operating voltage	10–20 V or higher	Few volts or <5 V	Reduced tunneling distance and optimized band alignment	Lower energy consumption and CMOS compatibility
Energy consumption	pJ‐nJ per operation	fJ‐pJ per operation	Lower voltage, current, and switching time	Orders‐of‐magnitude reduction in power
Endurance	10^3^–10^5^ cycles	Up to >10^6^ cycles	Reduced dielectric stress and defect generation at van der Waals interfaces	Improved cycling stability
Retention	≥10 years (industrial standard)	10^4^–10^5^ s (demonstrated; potentially higher)	Deep traps and reduced leakage via interface engineering	Potential for further improvement via trap engineering
Subthreshold swing	≥60 mV dec^−^^1^	< 60 mV dec^−^^1^ (with steep‐slope mechanisms)	Novel transport mechanisms (e.g., tunneling, NC effects)	Reduced switching energy and leakage
On/off ratio	10^6^–10^8^	> 10^9^	Strong gate control and reduced off‐state leakage	Enhanced switching performance
Interface trap density	Relatively high	Low (dangling‐bond‐free interfaces)	van der Waals interfaces with minimal defects	Improved reliability and uniformity
Injection mechanism	FN tunneling, hot‐carrier injection	FN tunneling, hot‐carrier injection, direct tunneling	Tunable barriers and multiple injection pathways	Greater flexibility in performance optimization
Integration density	High (3D NAND > 300 layers)	Atomic thickness ∼0.7 nm	Layer‐by‐layer stacking at atomic scale	Higher theoretical density limit
Integration maturity	Fully industrialized	Still under development	Challenges in large‐area synthesis and uniformity	Opportunities for heterogeneous integration
Application scope	Data storage (SSD, UFS, etc.)	Storage, neuromorphic computing, in‐memory computing, flexible electronics	Multifunctional device capability	Enables storage‐computing convergence

Table 1 compares the structural configurations and key performance metrics of conventional silicon‐based flash memory and 2D‐material‐based flash memory. In particular, programming speed can be improved from the conventional microsecond‐millisecond range to nanosecond or even subnanosecond regime, while operating voltages can be reduced from 10–20 V to only a few volts or below. In addition, energy consumption can be reduced by orders of magnitude. Reduced interface trap density and defect‐minimized van der Waals interfaces contribute to improved endurance and device reliability.

Meanwhile, the atomic thickness and stackability of 2D materials provide a natural pathway toward ultra‐high‐density integration [86]. Although current 2D flash devices still lag behind mature silicon technologies in terms of long‐term retention and large‐scale manufacturability, their intrinsic reconfigurability and heterointegration capability make them attractive for data storage, in‐memory computing, flexible electronics, and neuromorphic systems [87, 88, 89, 90, 91, 92]. In this sense, 2D materials do not merely extend flash memory; they redefine it as a multifunctional platform for post‐Moore information hardware.

## 3 Interface‐Controlled Design Principles for High‐Performance 2D Flash Memory

The optimization of 2D flash memory should not be viewed simply as an empirical pursuit of faster programming, lower operating power, longer retention, or higher endurance. Rather, these performance metrics originate from a common physical foundation. The central challenge is not the improvement of any single figure of merit, but the deliberate engineering of the interfacial energy landscape to enable coordinated optimization of speed, energy efficiency, retention, and reliability.

This perspective is critical because the long‐standing trade‐offs in conventional flash memory originate from the intrinsic coupling between charge injection and dielectric stress. In silicon‐based devices, programming and erasing rely on high‐field Fowler‐Nordheim tunneling or hot‐carrier injection. While these mechanisms enable nonvolatile operation, they also impose fundamental constraints: stronger electric fields accelerate charge transfer but inevitably increase energy dissipation, induce defect generation, and accelerate long‐term dielectric degradation [93, 94]. As a result, programming speed, power consumption, retention stability, and endurance remain intrinsically coupled.

2D materials provide a fundamentally different platform for overcoming these limitations. Their atomically thin bodies, dangling‐bond‐free surfaces, and highly tunable van der Waals heterostructures enable precise control over tunneling distance, barrier height, interfacial coupling, trap‐state distribution, and contact geometry. These degrees of freedom allow the charge‐storage process to be reconfigured at multiple levels—from injection pathways and local electric‐field distribution to the depth and spatial localization of trapped charges—thereby providing a pathway to partially decouple the conventional trade‐offs among speed, power consumption, retention, and endurance.

Within this unified framework of interface‐controlled charge programming, different performance metrics can be understood as outcomes of distinct but interconnected physical processes. Ultrafast operation is primarily governed by the reduction of effective injection barriers and the acceleration of carrier injection and trapping kinetics. Low‐power behavior emerges from the combined suppression of programming voltage and current together with shortened switching dynamics. Long data retention is dictated by the stability of deep trapping centers and the effective confinement of stored charges against leakage pathways. Achieving high endurance further requires that all these processes occur under minimized electrical and structural stress, thereby suppressing defect accumulation and interfacial degradation during repeated cycling.

Accordingly, this section discusses the optimization of 2D flash memory from four representative yet physically interconnected aspects: ultrafast programming/erasing, low‐power operation, long data retention, and improved reliability/endurance. Although these aspects are presented separately for clarity, they originate from a common design principle: the deliberate control of interfaces, energy barriers, trap states, and transport pathways to govern the full lifecycle of stored charges in 2D memory devices.

### 3.1 Ultrafast Programming: Engineering Injection Pathways and Energy Barriers

The programming speed of flash memory is determined by the efficiency of charge injection across energy barriers. In conventional flash, Fowler‐Nordheim tunneling and hot‐carrier injection dominate the programming process, both of which require high electric fields and are intrinsically constrained by the trade‐off between speed, power consumption, and reliability [95, 96, 97, 98]. Figure 5 illustrates that 2D materials enable a reconfiguration of charge injection pathways through atomically thin channels, van der Waals interfaces, and tunable contact geometries, allowing the transition from field‐driven tunneling to interface‐engineered, multiple injection mechanisms. As a result, programming speed can be dramatically improved from the microsecond regime to nanosecond and even subnanosecond timescales.

**FIGURE 5:**
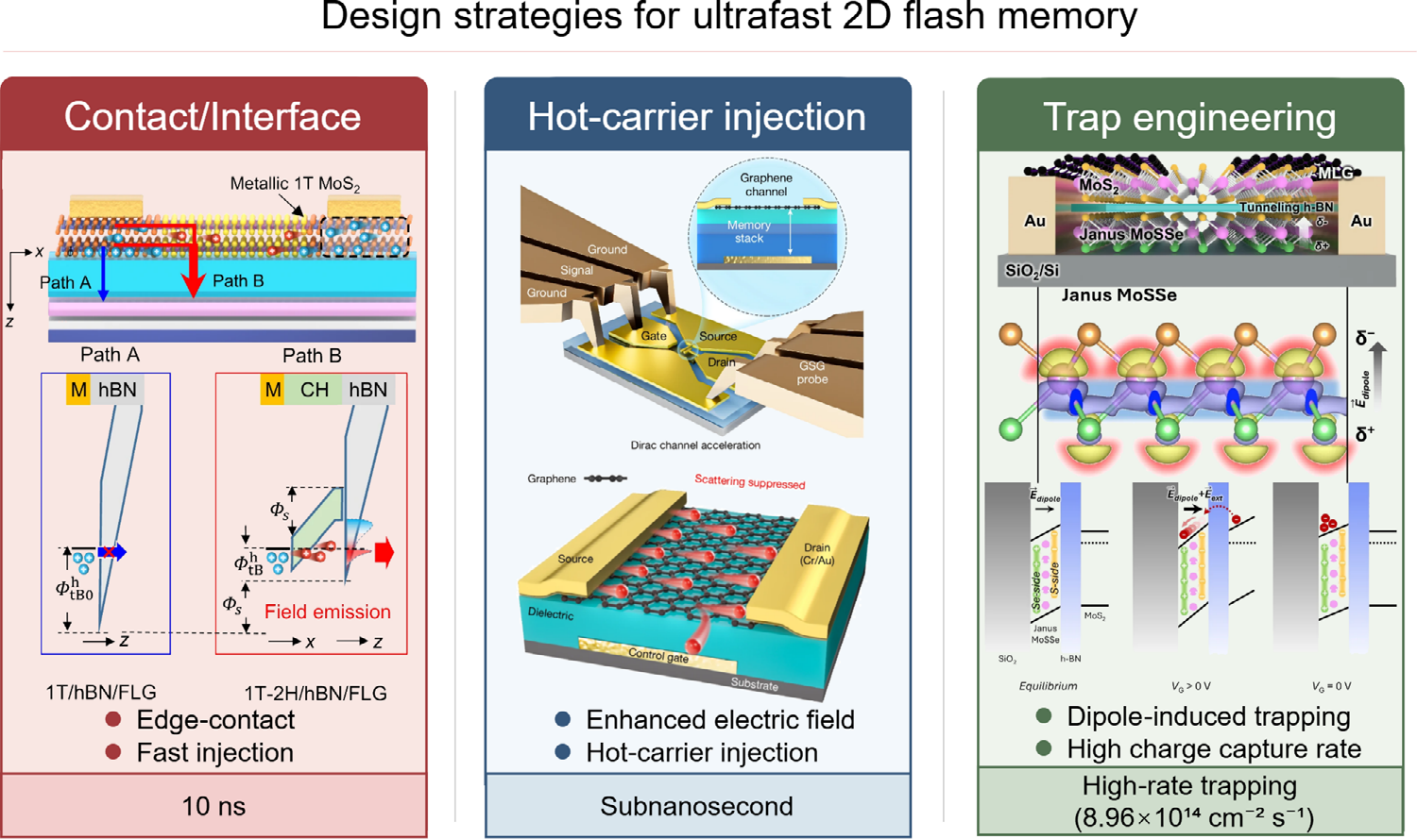
Interface‐controlled strategies for ultrafast 2D flash memory [34, 45, 69]. Reproduced under terms of the CC BY 4.0 license [34]. Copyright 2023, Springer Nature. Reproduced with permission [45]. Copyright 2025, Springer Nature. Reproduced under terms of the CC BY 4.0 license [69]. Copyright 2026, Springer Nature. Schematic summary of representative approaches for accelerating charge programming in 2D flash devices, including barrier engineering, interface‐controlled injection, direct tunneling, hot‐carrier injection, and accelerated trapping/detrapping processes. These strategies reduce the effective charge‐injection barrier and shorten the charge‐transfer pathway, enabling programming speeds from the nanosecond to subnanosecond regime.

#### 3.1.1 Barrier Modulation through Phase‐Engineered Contacts and van der Waals Interfaces

Reducing the effective charge‐injection barrier is one of the most direct routes toward ultrafast flash operation. Yu et al. [34] demonstrated this concept by integrating phase‐engineered contacts with van der Waals heterostructures in a MoS_2_/hBN/few‐layer graphene flash device, achieving a programming speed of approximately 10 ns. In this design, hBN serves as the tunneling layer, few‐layer graphene as the floating gate, and locally lithiated 1T‐LixMoS_2_ forms a metallic edge contact to semiconducting 2H‐MoS_2_ (Figure 6a).

**FIGURE 6:**
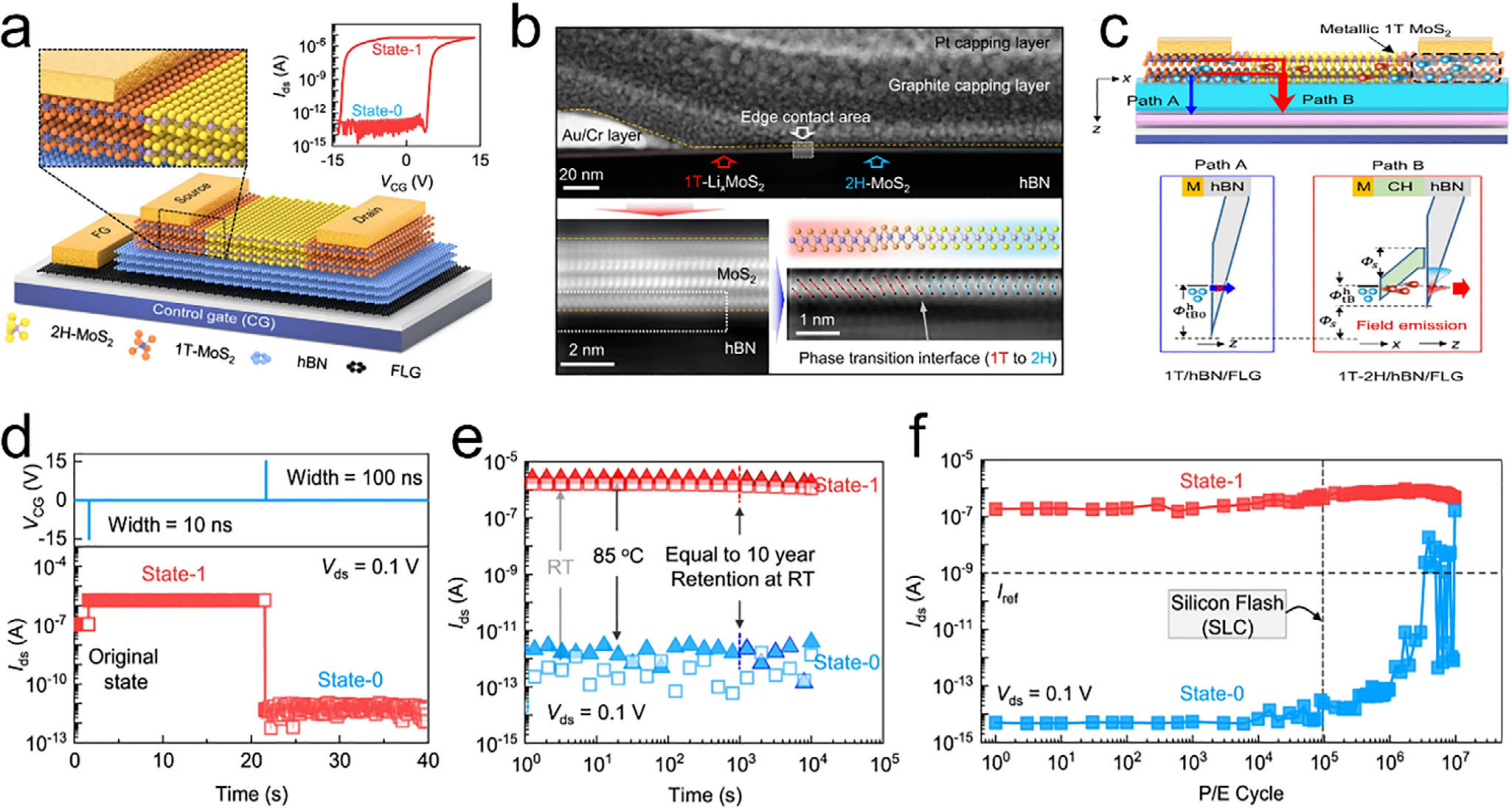
Phase‐engineered edge‐contacted MoS_2_/hBN/few‐layer graphene flash memory for ultrafast programming [34]. Reproduced under terms of the CC BY 4.0 license [34]. Copyright 2023, Springer Nature. (a) Device schematic of the MoS_2_/hBN/few‐layer graphene flash memory with a metallic 1T‐LixMoS_2_ edge contact. (b) HAADF‐STEM images confirming the structural integrity of the phase‐engineered edge‐contact region. (c) Schematic illustration of the carrier‐injection pathway enabled by the lateral 1T/2H phase boundary. (d) Nanosecond‐scale programming/erasing characteristics under short voltage pulses. (e) Retention characteristics of the programmed and erased states. (f) Endurance performance under repeated programming/erasing cycles.

The HAADF‐STEM image in Figure 6b confirmed the structural integrity of the phase‐engineered edge‐contact region. Unlike conventional top metal contacts, which often induce lattice damage and strong Fermi‐level pinning in 2D semiconductors, the phase‐engineered edge contact avoids deposition‐induced disorder and enables dynamic gate modulation of the Schottky barrier. Figure 6c illustrates that this contact geometry reconstructs the injection pathway from a vertical metal/semiconductor crossing into a lateral transition through the phase boundary, substantially enhancing hot‐carrier injection efficiency without dramatically increasing stress on the tunneling dielectric. As a result, the device achieves nanosecond‐scale programming/erasing speed (Figure 6d) while maintaining excellent retention (Figure 6e) and endurance (Figure 6f). This study shows that contact and interface co‐engineering can reconfigure the speed‐reliability balance in 2D flash memory.

A related strategy was reported by Wu et al. [99], who constructed a vertically stacked InSe/hBN/multilayer graphene floating‐gate memory (Figure 7a) and achieved programming/erasing in 21 ns. The key to this performance lies in the formation of atomically clean and nearly defect‐free interfaces, as confirmed by high‐resolution HAADF‐STEM characterization in Figure 7b. Such pristine van der Waals interfaces reduce interfacial scattering and barrier inhomogeneity, thereby improving the uniformity and efficiency of charge injection. The resulting devices combine nanosecond‐scale programming (Figure 7c) with excellent nonvolatile retention (Figure 7d), high endurance (Figure 7e), and a large on/off ratio. Together, these studies demonstrate that ultrafast operation in 2D flash memory is not merely a consequence of thinner channels, but of precisely engineered injection barriers and interfacial coupling.

**FIGURE 7:**
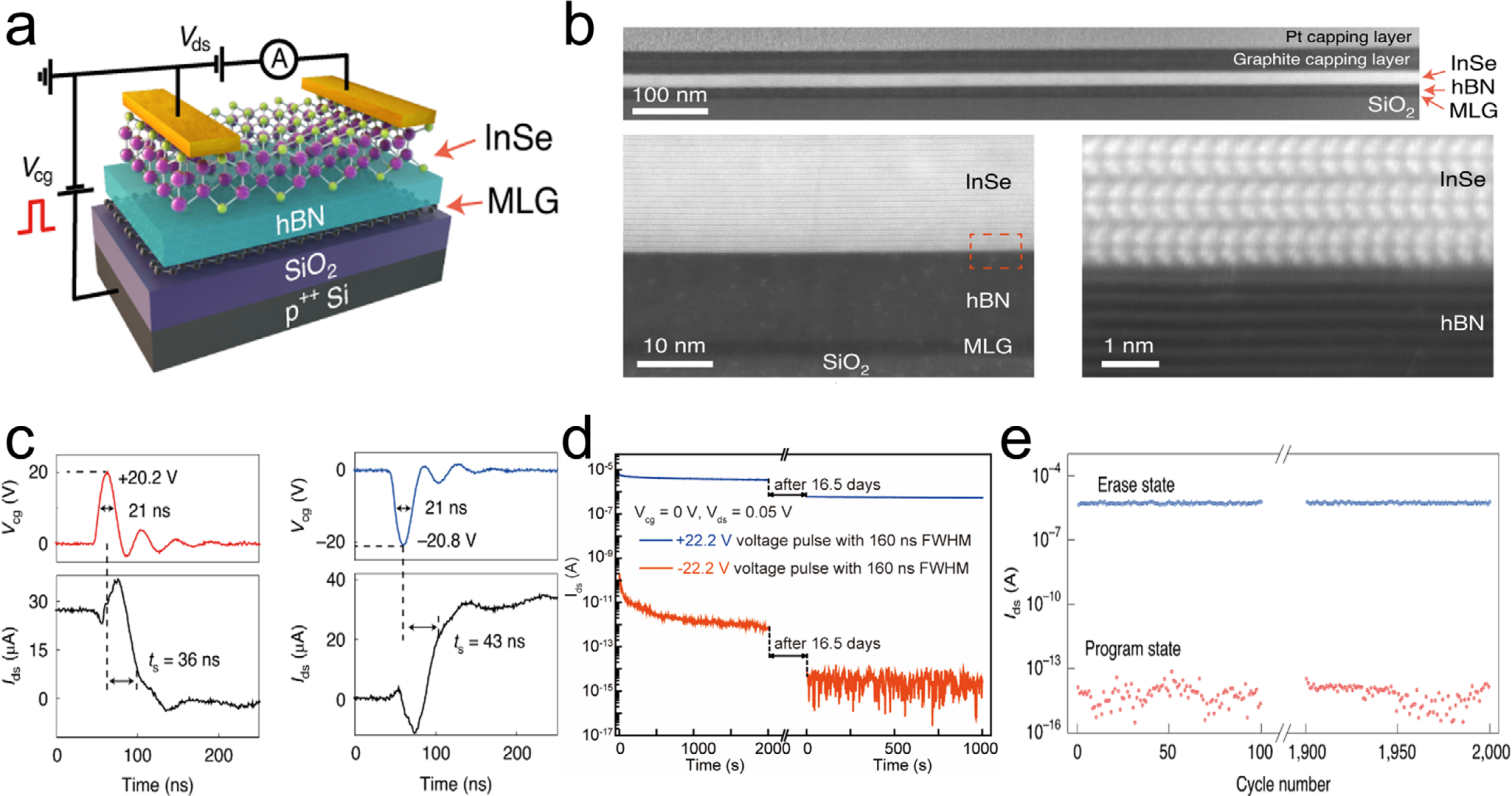
Ultrafast InSe/hBN/multilayer graphene van der Waals floating‐gate flash memory [99]. Reproduced with permission [99]. Copyright 2021, Springer Nature. (a) Schematic of the vertically stacked InSe/hBN/multilayer graphene floating‐gate memory. (b) Cross‐sectional HAADF‐STEM images showing clean and atomically sharp van der Waals interfaces. (c) Nanosecond‐scale programming/erasing response of the device. (d) Retention characteristics showing nonvolatile charge storage. (e) Endurance performance during repeated programming/erasing cycles.

#### 3.1.2 2D‐Enhanced Hot‐Carrier Injection and Direct Tunneling

Another effective route toward ultrafast operation is to exploit the unique electric‐field distribution and carrier dynamics of atomically thin channels. Xiang et al. [45] reported a subnanosecond flash memory based on a graphene channel coupled to a storage stack, in which 2D‐enhanced hot‐carrier injection operates synergistically with direct tunneling. Figure 8a–c shows the device structure and the corresponding graphene‐ and WSe_2_‐channel transistor configurations.

**FIGURE 8:**
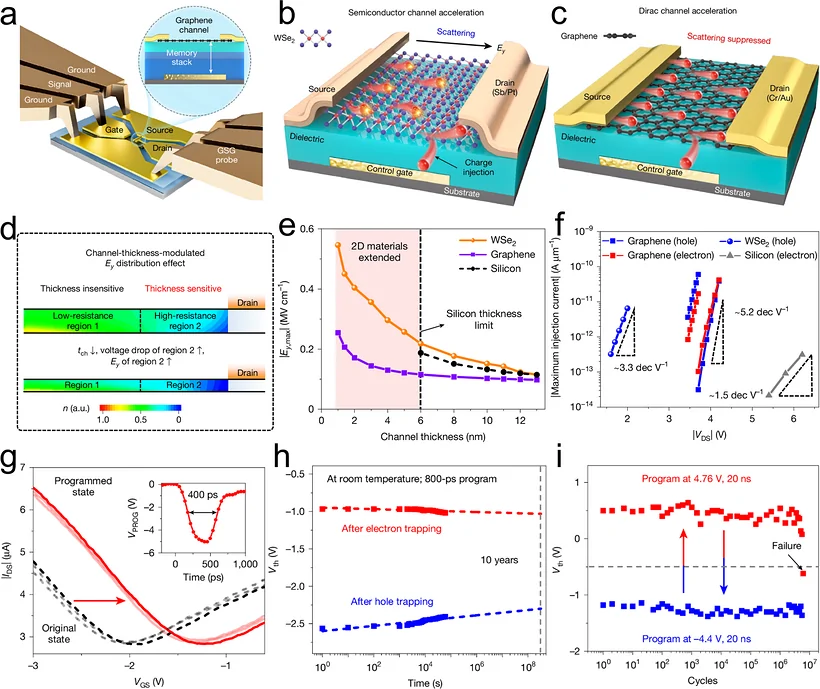
2D‐enhanced hot‐carrier injection and direct tunneling for subnanosecond flash programming [45]. Reproduced with permission [45]. Copyright 2025, Springer Nature. (a) Schematic of the graphene‐channel flash memory device and pulse‐testing configuration. (b) Schematic of the WSe_2_‐channel transistor used for comparison. (c) Schematic of the graphene‐channel transistor. (d) Simulated channel‐thickness‐dependent lateral electric‐field distribution. (e) Maximum lateral electric field as a function of channel thickness for WSe_2_, graphene, and silicon channels. (f) Maximum injection current as a function of electric field for different channel materials. (g) Subnanosecond programming behavior under short voltage pulses. (h) Retention performance of the programmed memory states. (i) Endurance characteristics over repeated programming/erasing cycles.

Compared with conventional silicon channels, atomically thin 2D channels can concentrate the lateral electric field near the drain more strongly, particularly as the channel thickness decreases (Figure 8d). This enhanced field accelerates carriers more efficiently and increases the probability of injection into the storage layer. Figure 8e,f confirms that this effect is particularly pronounced in graphene because of its high carrier concentration and weak scattering, enabling both electrons and holes to acquire sufficient energy over a wider spatial region than in silicon or semiconducting 2D channels such as WSe_2_.

As a result, the graphene‐based device achieves programming in approximately 400 ps at around 5 V (Figure 8g), breaking the conventional nanosecond barrier of flash memory. Importantly, this ultrafast speed does not come at the cost of nonvolatile functionality: the device still exhibits retention beyond 10^4^ s (Figure 8h) and endurance above 5.5 × 10^6^ cycles (Figure 8i). The same design principle was also shown to be applicable to WSe_2_ channels, indicating that 2D‐enhanced hot‐carrier injection is not limited to graphene, but represents a more general mechanism for ultrafast flash design in 2D systems.

#### 3.1.3 Accelerated Trapping and Detrapping through Trap Engineering

Beyond injection efficiency, the kinetics of charge trapping and release in the storage layer also play a critical role in determining programming speed. Ko et al. [69] introduced monolayer Janus MoSSe as a charge‐trapping layer in a vertical MLG/MoS_2_/ultrathin hBN/Janus MoSSe heterostructure (Figure 9a). Because of its intrinsic out‐of‐plane dipole moment, Janus MoSSe provides deep and stable trapping sites that strongly confine charges, while the ultrathin hBN tunneling layer shortens the tunneling distance and accelerates charge transfer.

**FIGURE 9:**
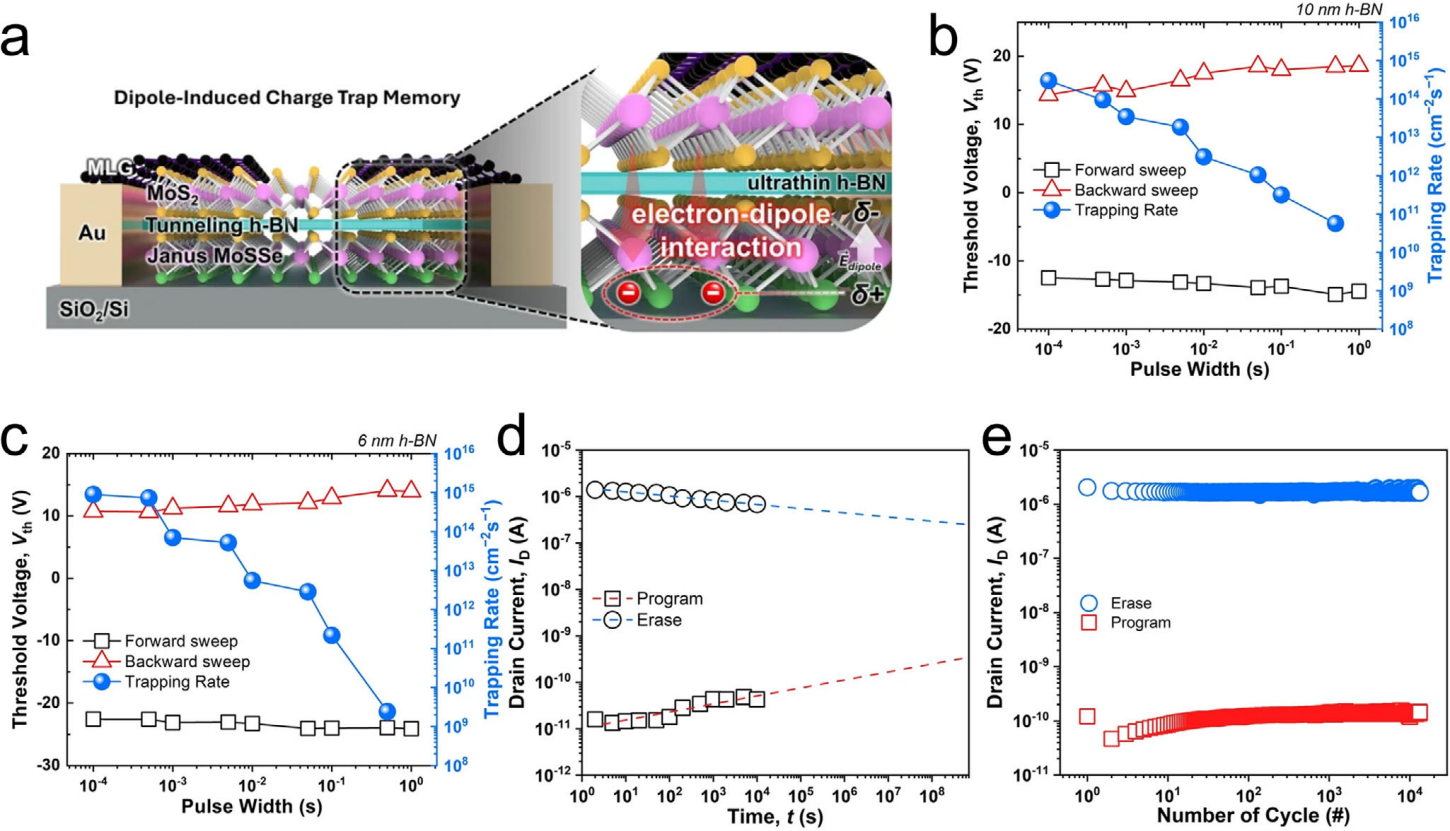
Dipole‐assisted charge‐trap flash memory based on Janus MoSSe and ultrathin hBN [69]. Reproduced under terms of the CC BY 4.0 license [69]. Copyright 2026, Springer Nature. (a) Schematic of the multilayer graphene/MoS_2_/hBN/Janus MoSSe charge‐trap memory. (b,c) Threshold‐voltage shifts and calculated charge‐trapping rates for devices with different hBN tunneling‐layer thicknesses. (d) Retention characteristics showing stable charge confinement. (e) Endurance performance under repeated programming/erasing operation.

This design enables a balance that is difficult to achieve in conventional systems: fast tunneling injection through a thin dielectric together with stable charge storage enabled by dipole‐induced trap confinement. Compared with devices using thicker hBN, thinner hBN layers produce significantly faster trapping rates and larger memory windows (Figure 9b,c), while retention (Figure 9d) and endurance (Figure 9e) remain robust. This work highlights that trap engineering in 2D flash memory is not only about long retention, but also about accelerating the full trapping/detrapping dynamics, thereby contributing directly to high‐speed operation.

### 3.2 Low‐Power Operation: Minimizing Voltage, Current, and Switching Time

Reducing power consumption in flash memory requires simultaneous optimization of programming voltage, current, and switching time. In conventional flash devices, the high electric fields required for Fowler‐Nordheim tunneling lead to substantial energy dissipation and poor compatibility with low‐voltage CMOS circuits. Figure 10 illustrates that 2D flash memory leverages heterostructure engineering, interface modulation, and contact design to enable efficient charge programming under significantly reduced electric fields. By lowering injection barriers, suppressing leakage currents, and shortening programming time, these strategies enable orders‐of‐magnitude reductions in operating energy.

**FIGURE 10:**
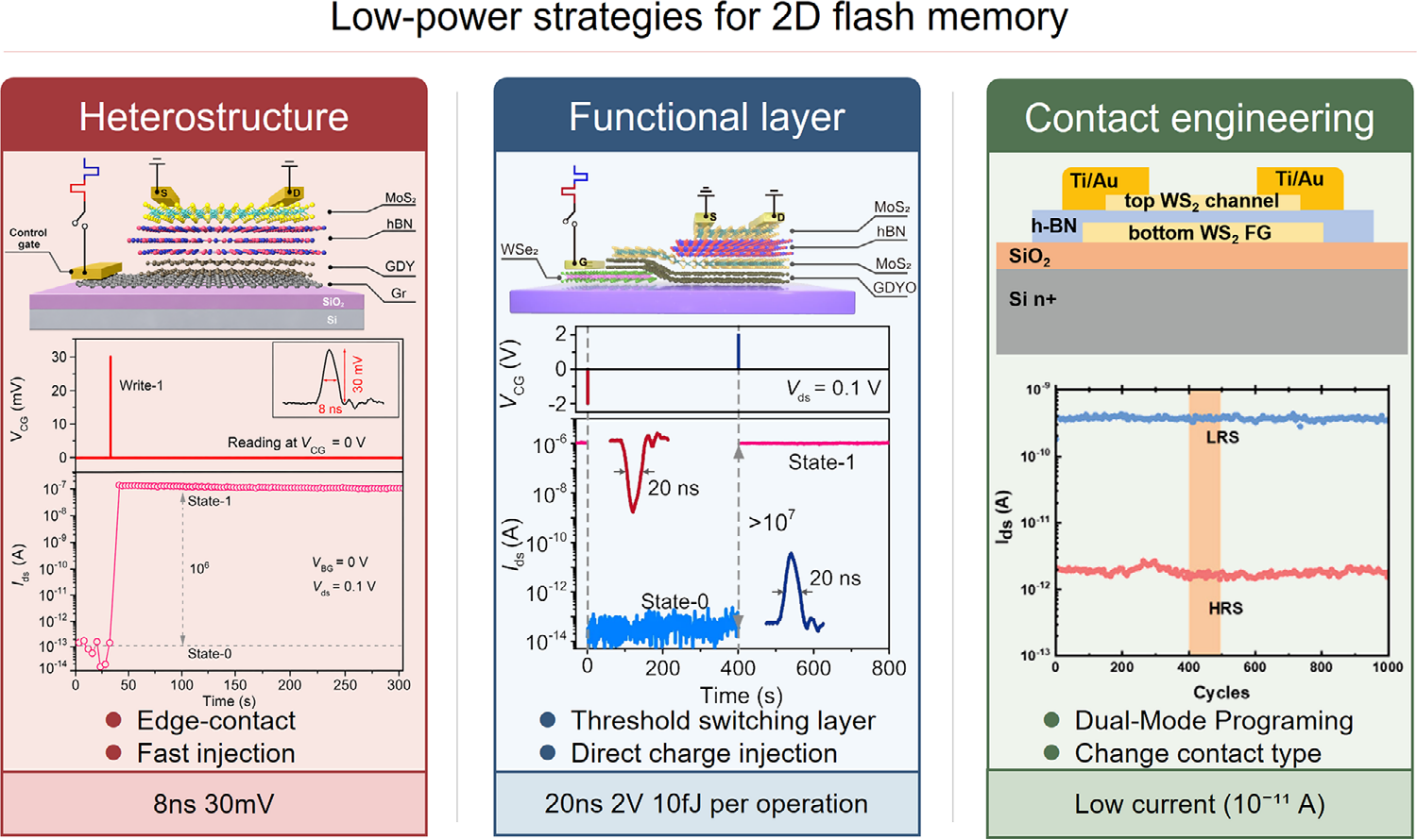
Structural strategies for low‐power 2D flash memory [100, 101, 102]. Reproduced with permission [100]. Copyright 2021, Wiley. Reproduced under terms of the CC BY 4.0 license [101]. Copyright 2022, Springer Nature. Reproduced under terms of the CC BY 4.0 license [102]. Copyright 2024, Wiley. Schematic summary of representative approaches for reducing programming energy in 2D flash devices through heterostructure design, functional‐layer engineering, contact modulation, and band‐alignment optimization. By lowering programming voltage, suppressing leakage current, and shortening switching time, these strategies enable energy‐efficient nonvolatile operation.

#### 3.2.1 Heterostructure and Functional‐Layer Co‐Engineering

One effective strategy for low‐power programming is to optimize the coupling among channel, tunneling layer, storage layer, and gate through heterostructure and functional‐layer design. Zhang's group demonstrated a MoS_2_/hBN/ graphdiyne (GDY) /graphene van der Waals memory in which GDY directly coupled to a graphene gate acts as a charge‐storage layer, forming a semi‐floating‐gate architecture (Figure 11a) [100]. Because the GDY/graphene stack provides favorable band alignment, the injection barrier is lowered and fast programming can be achieved at ultralow voltages. The device operates with write pulses of only 30 mV (Figure 11b) and −0.5 V for erase pulses (Figure 11c), with fast and energy‐efficient charge programming.

**FIGURE 11:**
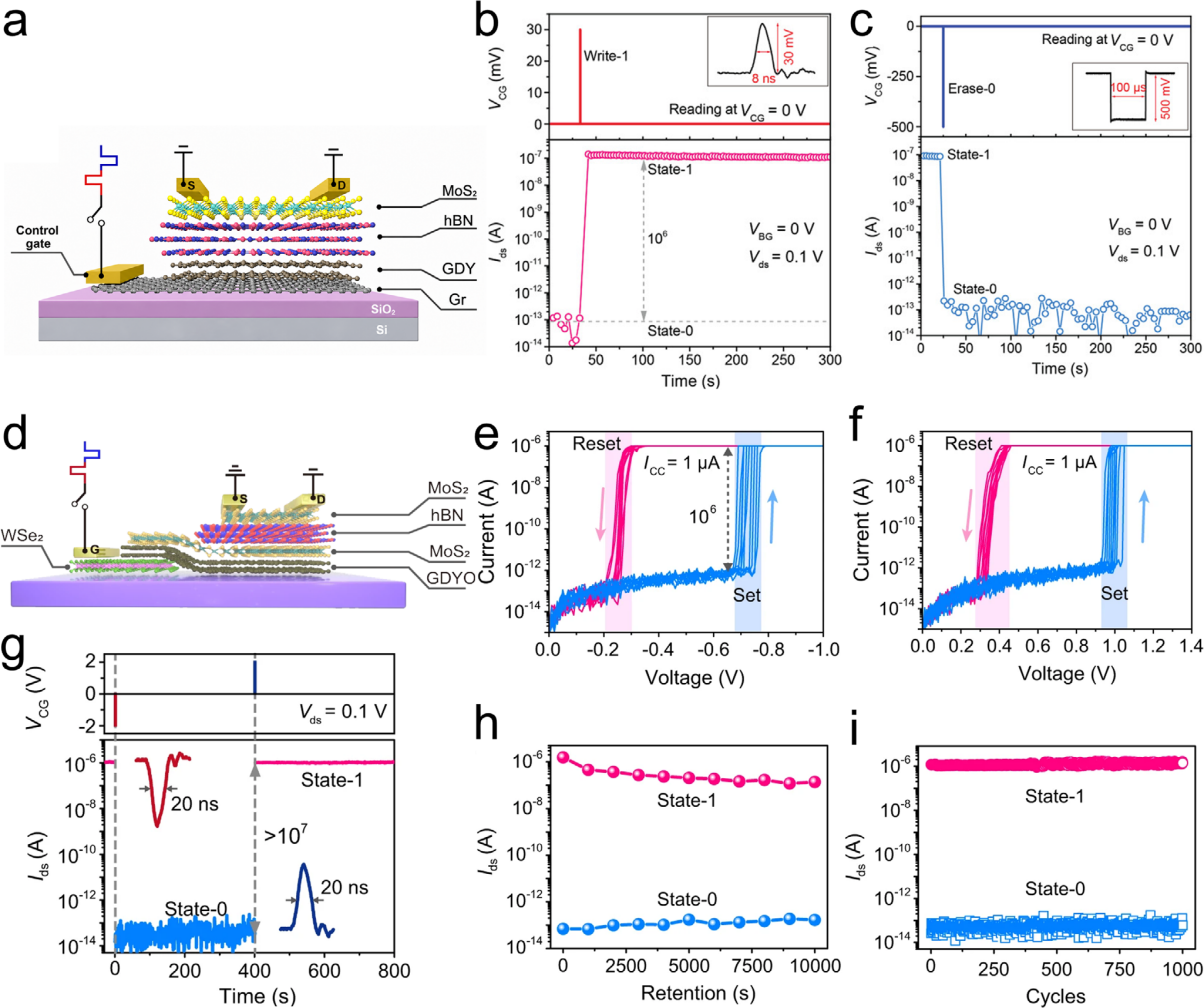
Low‐power 2D flash memory enabled by heterostructure and functional‐layer co‐engineering [100, 101]. Reproduced with permission [100]. Copyright 2021, Wiley. Reproduced under terms of the CC BY 4.0 license [101]. Copyright 2022, Springer Nature. (a) Schematic of the MoS_2_/hBN/graphdiyne/graphene nonvolatile memory device. (b) Ultralow‐voltage write operation enabled by efficient charge injection. (c) Erase operation of the same device. (d) Schematic of the MoS_2_/hBN/MoS_2_/GDYO/WSe_2_ nonvolatile memory incorporating a threshold‐switching layer. (e, f) Threshold‐switching behavior under negative and positive bias, respectively. (g) Fast programming and erasing characteristics under short voltage pulses. (h) Retention performance of the programmed states. (i) Endurance performance during repeated operation.

The same group further introduced a threshold‐switching layer based on graphdiyne oxide between the control gate and floating gate (Figure 11d) [101]. Instead of relying solely on Fowler‐Nordheim tunneling, this architecture exploits a transient low‐resistance state in the threshold‐switching layer under programming pulses, enabling direct and rapid charge injection from the control gate into the floating gate. As shown in Figure 11e,f, once the pulse is removed, the layer returns to its high‐resistance state, suppressing back leakage and preserving the stored charge. This design achieves programming/erasing with a 20 ns voltage pulse at only 2 V (Figure 11g), with an energy cost as low as 10 fJ per operation, while retaining robust nonvolatile performance (Figure 11h,i). These studies demonstrate that dynamic functional layers can reduce the voltage and energy required for programming without sacrificing retention.

#### 3.2.2 Contact Engineering for Low‐Energy Operation

In addition to barrier and layer design, contact engineering provides another route to low‐power flash operation by directly modulating the programming current. Zhang et al. [102] reported a WS_2_/hBN/WS_2_ van der Waals memory (Figure 12a) in which the effective contact type at the metal/WS_2_ interface is tuned through gate bias. Under positive gate bias, the carrier concentration in the WS_2_ channel increases, narrowing the Schottky barrier and allowing near‐ohmic transport under sufficiently high source‐drain bias (Figure 12b). Under negative gate bias, the carrier concentration is depleted and the barrier becomes wider and higher, restoring a more strongly rectifying Schottky‐like behavior (Figure 12c).

**FIGURE 12:**
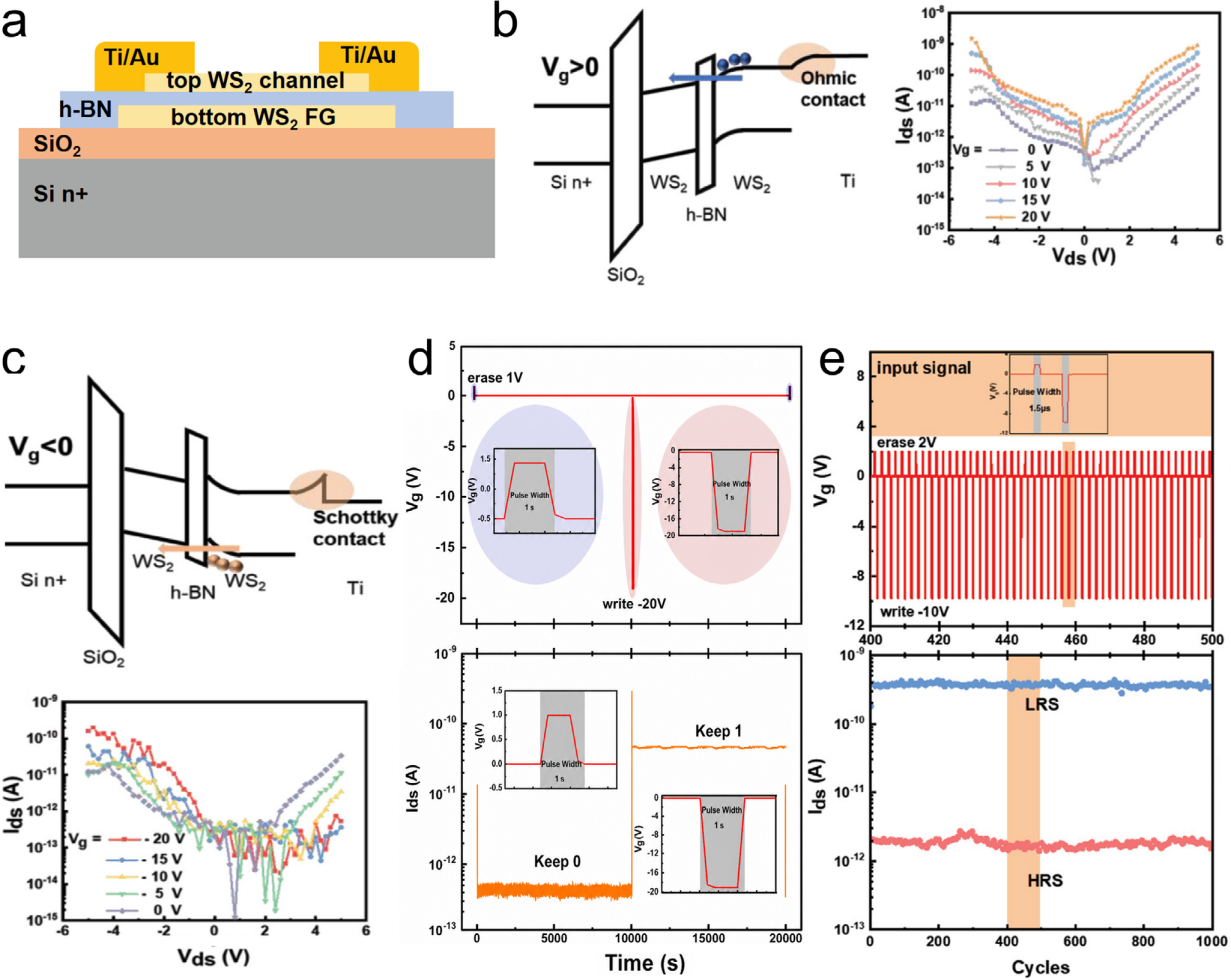
Low‐power flash memory enabled by gate‐tunable contact engineering [102]. Reproduced under terms of the CC BY 4.0 license [102]. Copyright 2024, Wiley. (a) Schematic of the WS_2_/hBN/WS_2_ van der Waals memory device. (b) Gate‐tunable quasi‐ohmic contact formed under positive gate bias, enabling efficient charge injection. (c) Gate‐tunable Schottky contact formed under negative gate bias, suppressing current and leakage. (d) Retention characteristics of the device. (e) Endurance performance during repeated programming/erasing cycles.

This gate‐tunable contact behavior allows the same device to switch between a high‐current, quasi‐ohmic programming state and an ultralow‐current Schottky‐dominated state. As a result, the programming current can be reduced to approximately 10^−11^ A while maintaining stable retention (Figure 12d) and endurance (Figure 12e). This work shows that low‐power 2D flash memory can be achieved by reducing voltage and actively suppressing programming current through contact‐mode control.

### 3.3 Data Retention: Stabilizing Charge Storage through Traps and Interfaces

Data retention in flash memory depends on the long‐term stability of stored charges. As devices are scaled down, charge leakage, trap instability, and interface degradation become increasingly problematic. Figure 13 illustrates that retention in 2D flash systems can be enhanced through the design of deep and stable trapping centers, the minimization of leakage pathways via high‐quality van der Waals interfaces, and the strengthening of electrostatic confinement. These strategies allow 2D flash memories to approach long‐term retention levels that begin to rival mature silicon technologies.

**FIGURE 13:**
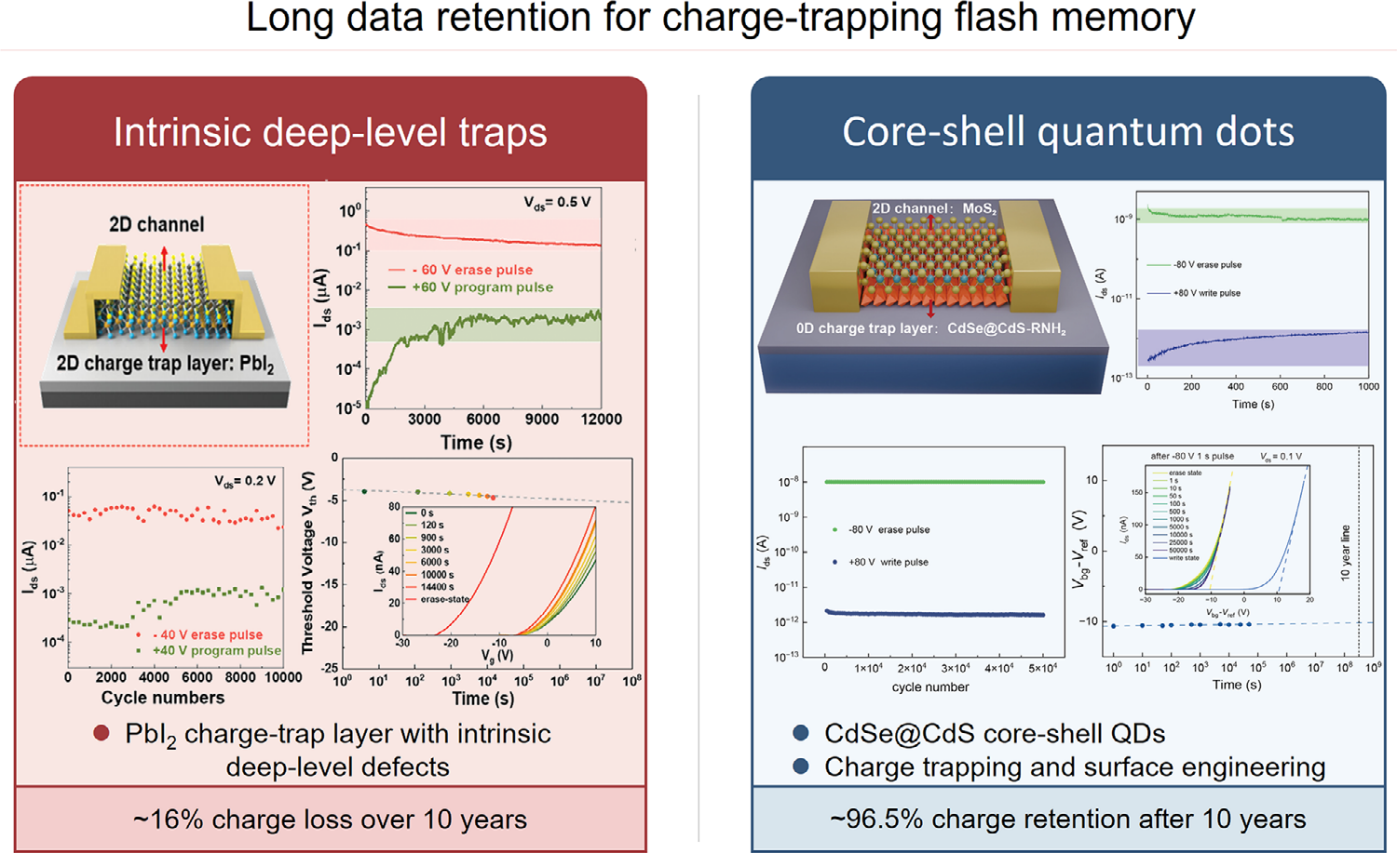
Design concepts for long‐retention 2D flash memory [103, 104]. Reproduced with permission [103]. Copyright 2023, Wiley. Reproduced with permission [104]. Copyright 2026, Elsevier. Schematic summary of representative strategies for stabilizing stored charges in 2D charge‐trap memories, including the introduction of deep trapping centers, suppression of interfacial defects, reduction of leakage pathways, and enhancement of electrostatic confinement. These strategies are essential for improving long‐term nonvolatile retention while maintaining efficient programming.

#### 3.3.1 Deep‐Level Traps and Leakage Suppression for Retention Enhancement

A direct route to long retention is to employ charge‐storage layers with intrinsic deep‐level defects. Liu et al. [103] reported a van der Waals charge‐trap memory using PbI_2_ as a single trapping medium (Figure 14a). Unlike conventional trap layers that rely on artificial defects or high‐k oxides, PbI_2_ naturally contains abundant iodine‐vacancy‐related trapping sites because of the low formation energy of iodine vacancies. These intrinsic traps not only provide high trapping density, but also reside relatively deep in energy, which greatly suppresses charge loss.

**FIGURE 14:**
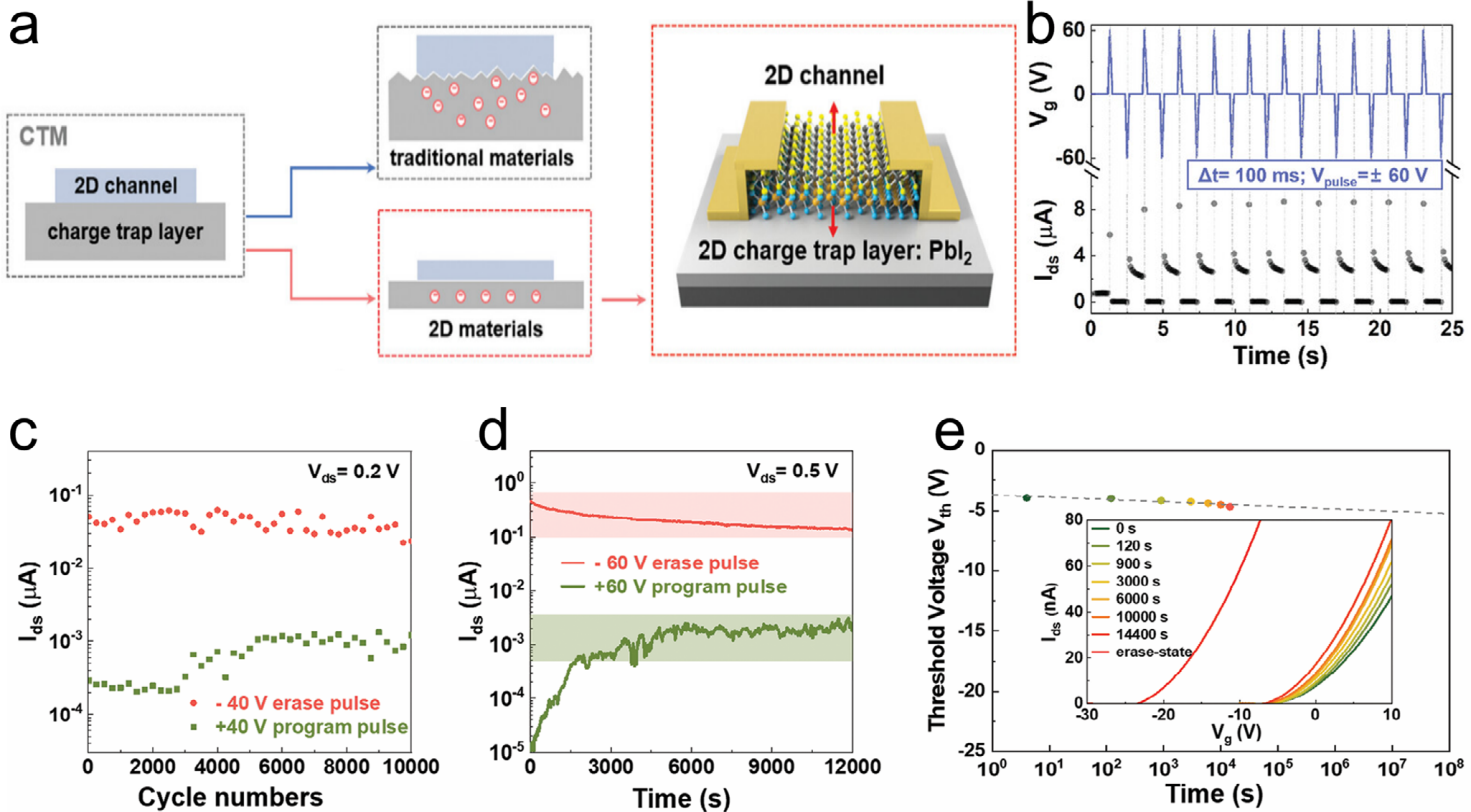
PbI_2_‐based 2D charge‐trap memory with intrinsic deep‐level defect states [103]. Reproduced with permission [103]. Copyright 2023, Wiley. (a) Schematic of the PbI_2_‐based charge‐trap memory device. (b) Stable programming/erasing characteristics. (c) Endurance performance under repeated cycling. (d) Retention characteristics showing slow threshold‐voltage drift. (e) Threshold‐voltage evolution and projected long‐term retention behavior, indicating effective charge confinement by intrinsic deep‐level traps.

The resulting devices exhibit stable and repeatable programming/erasing operation (Figure 14b), excellent endurance (Figure 14c), and very slow threshold‐voltage drift over retention testing (Figure 14d). The device experimentally demonstrated retention beyond 1.2 × 10^4^ s, from which the original study extrapolated a charge loss of approximately 16% after 10 years (Figure 14e). This work demonstrates that intrinsic defect‐rich 2D materials can serve as effective deep‐level trapping layers for long‐retention flash memory without introducing excessive interfacial complexity.

#### 3.3.2 Coupling with Core–Shell Quantum Dots and Interface Defect Suppression

Retention can also be improved by minimizing interfacial charge loss through nanostructure engineering. Qiu et al. [104] developed a monolayer MoS_2_ charge‐trap memory using CdSe@CdS‐RNH_2_ core‐shell quantum dots as the charge‐storage layer (Figure 15a). In this design, the CdSe core is fully encapsulated by a CdS shell, and the surface is passivated with electrochemically inert ligands, reducing surface defects and suppressing nonradiative charge loss.

**FIGURE 15:**
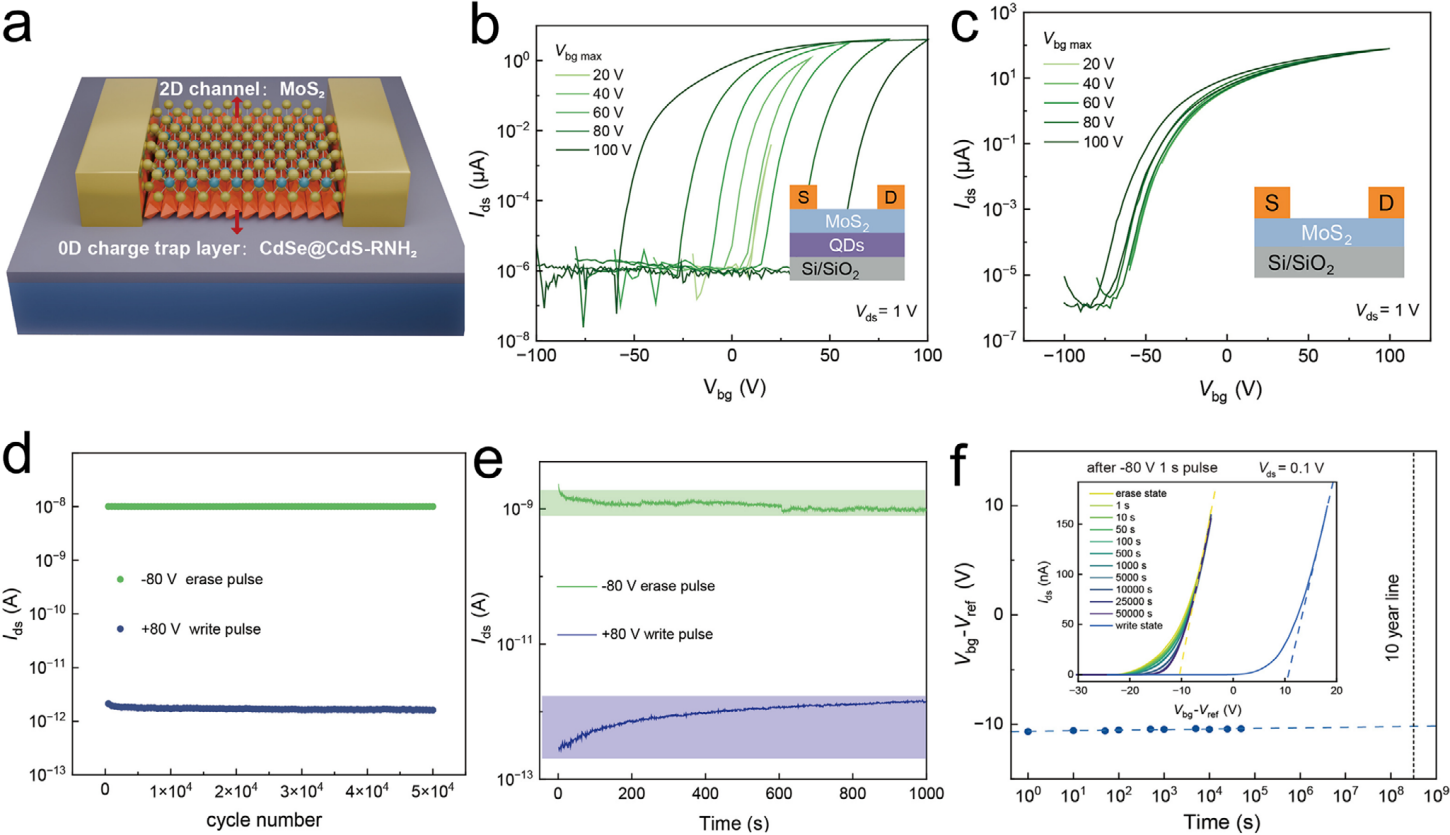
Monolayer MoS_2_ charge‐trap memory coupled with CdSe@CdS core‐shell quantum dots [104]. Reproduced with permission [104]. Copyright 2026, Elsevier. (a) Schematic of the monolayer MoS_2_ charge‐trap memory incorporating CdSe@CdS‐RNH_2_ quantum dots as the charge‐storage layer. (b, c) Transfer characteristics and memory‐window enhancement after introducing the quantum‐dot trapping layer. (d) Endurance performance under repeated programming/erasing cycles. (e) Retention characteristics of the programmed states. (f) Threshold‐voltage evolution and projected long‐term charge retention enabled by quantum‐dot trapping and surface passivation.

Figure 15b,c confirms that the substantial enlargement of the memory window upon introducing the quantum‐dot layer indicates the formation of abundant and stable charge‐trapping sites. The device maintains stable switching over more than 5 × 10^4^ cycles (Figure 15d) and exhibits very small threshold drift during long‐term retention measurements (Figure 15e). Retention measurements were performed over 5 × 10^4^ s, and extrapolation of the measured decay behavior suggested that approximately 96.5% of the stored charge could be retained after 10 years (Figure 15f). This result highlights that quantum‐dot coupling and surface passivation offer an effective route to simultaneously increase trapping density and suppress interfacial leakage in 2D charge‐trap memories.

### 3.4 Reliability and Endurance: Mitigating Degradation through Interface and Process Control

Endurance reflects the ability of flash memory to withstand repeated programming/erasing operations without severe degradation. In conventional devices, repeated high‐field operation accumulates electrical and structural stress in the tunneling dielectric, charge‐storage layer, and interfaces, leading to defect generation, trap evolution, and irreversible charge migration [105, 106]. The resulting degradation causes memory‐window collapse, threshold drift, and reduced precision in multilevel or analog operation. In applications such as in‐memory computing and neuromorphic systems, which require frequent and repeated weight updates, endurance becomes especially critical [107].

2D flash memory possesses intrinsic advantages for improving endurance. On the one hand, van der Waals interfaces contain fewer dangling bonds and can therefore reduce interfacial degradation during cycling. On the other hand, tunable contacts, ultrathin channels, and engineered storage layers allow charge programming to occur under lower electrical stress, slowing the accumulation of damage. As a result, endurance exceeding 10^6^ cycles has already been reported in several 2D flash systems.

More importantly, endurance in 2D flash memory is not simply a device reliability metric. It is also a prerequisite for practical deployment in data‐intensive and adaptive computing systems. High endurance ensures stable weight updating in analog arrays, reliable long‐term plasticity in neuromorphic applications, and sustained operation under repeated computation‐storage cycles. In this sense, endurance is a bridge between device physics and system‐level functionality.

## 4 In‐Memory Computing Architectures Based on 2D Flash Memory

Conventional computing systems are built upon the von Neumann architecture, in which memory and processing units are physically separated. The frequent movement of data between these units gives rise to substantial latency and energy consumption, which has become a major bottleneck for data‐intensive applications such as artificial intelligence and big‐data analytics. In‐memory computing addresses this challenge by performing computation directly within memory arrays, thereby minimizing data transfer at the architectural level and substantially improving both energy efficiency and throughput [108, 109, 110].

In this context, flash memory represents a particularly attractive platform for in‐memory computing owing to its high storage density, nonvolatility, low cost, and compatibility with CMOS technology. Its core operating principle lies in the charge‐controlled modulation of transistor threshold voltage: by injecting or releasing charges, flash cells can store data nonvolatilely while mapping programmable conductance states to synaptic weights in neural networks. This capability enables core operations such as vector‐matrix multiplication to be executed directly inside memory arrays, fundamentally alleviating the data‐movement bottleneck of von Neumann systems [88, 111, 112, 113, 114, 115, 116].

Compared with conventional silicon flash, 2D flash memory offers distinct advantages for in‐memory computing. As illustrated in Figure 16, its atomically thin channels, highly tunable interfaces, and reconfigurable heterostructures enable fine control over analog conductance states, thereby providing a promising basis for high‐precision weight representation [117]. Moreover, the multifunctionality of 2D material systems makes it possible to integrate sensing, storage, linear computing, and nonlinear activation within the same device platform [118].

**FIGURE 16:**
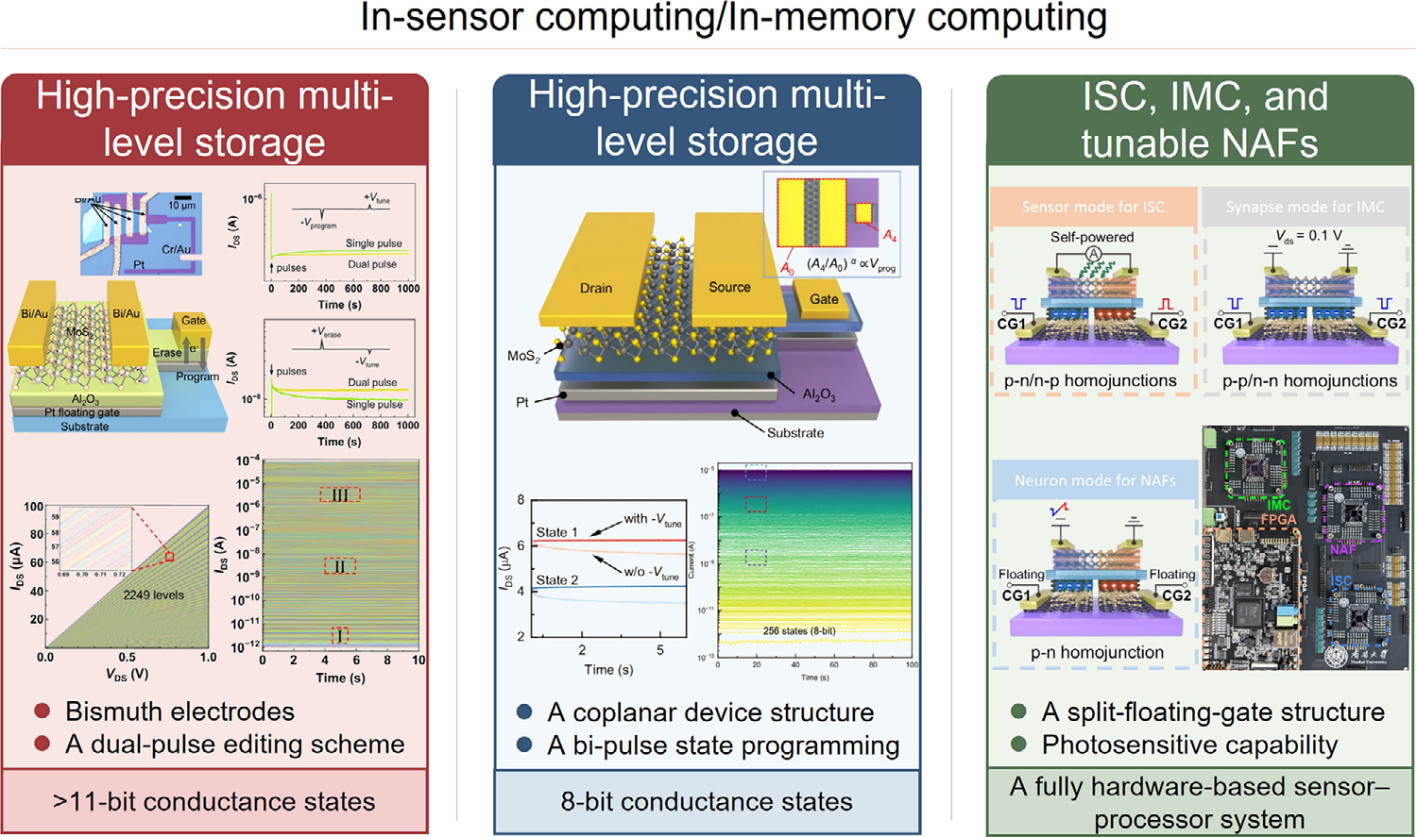
Flash‐memory‐based in‐memory computing using programmable conductance states [35, 36, 119]. Reproduced with permission [35]. Copyright 2025, Springer Nature. Reproduced under terms of the CC BY 4.0 license [36]. Copyright 2026, Springer Nature. Reproduced with permission [119]. Copyright 2025, Springer Nature. Conceptual illustration showing how nonvolatile threshold‐voltage or conductance states in flash memory cells can be mapped to synaptic weights. By executing vector‐matrix multiplication directly within memory arrays, flash‐memory‐based in‐memory computing reduces data transfer between memory and processing units and improves the energy efficiency of data‐centric computation.

### 4.1 High‐Precision Multilevel Storage

High‐precision multilevel storage is a prerequisite for analog in‐memory computing. The number of stable and distinguishable conductance states in a flash device directly determines the achievable weight precision and ultimately affects the inference accuracy of hardware neural‐network systems. In general, achieving high‐precision multilevel storage requires a large dynamic range, strong state stability, and low noise (Figure 17a). Wang et al. [35] addressed this challenge by combining barrier‐free bismuth contacts with a dual‐pulse editing scheme in a 2D floating‐gate memory (Figure 17b). The optimized gate‐injection architecture exhibited an on/off ratio of 10^8^, significantly reduced current noise, and improved state stability, thereby satisfying the key conditions for high‐resolution multilevel programming. As a result, the experimentally measured device achieved 2249 distinguishable conductance states and retained more than 11‐bit storage capacity even at 85°C (Figure 17c,d). Importantly, these multilevel characteristics were not obtained at the expense of dynamic performance or reliability: the device maintained a programming/erasing speed in approximately 230 ns (Figure 17e,f), while also showing retention beyond 10^4^ s (Figure 17g) and endurance above 10^5^ cycles (Figure 17h). This work highlights that high‐precision analog weight storage in 2D flash memory requires coordinated optimization of contact engineering, noise suppression, and pulse‐programming protocols.

**FIGURE 17:**
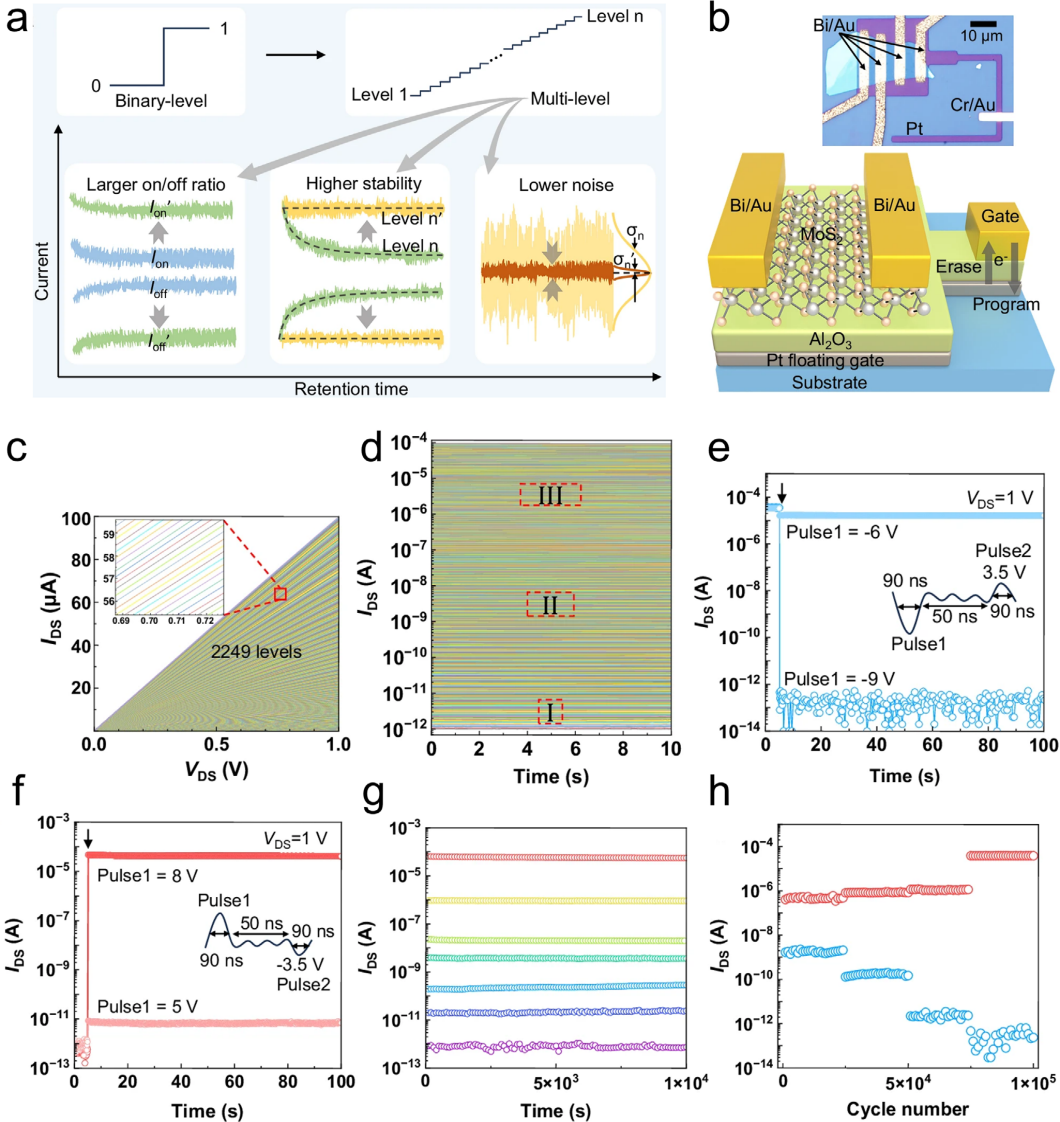
High‐precision multilevel storage in a 2D floating‐gate memory [35]. Reproduced with permission [35]. Copyright 2025, Springer Nature. Reproduced with permission [35]. Copyright 2025, Springer Nature. (a) Three key requirements for increasing the number of stable storage states in floating‐gate memories: large dynamic range, low noise, and strong state stability. (b) Schematic of the gate‐injection 2D floating‐gate memory device. (c) Output characteristics showing 2249 distinguishable conductance states. (d) Time‐dependent current characteristics of representative multilevel states. (e) Rapid programming enabled by the dual‐pulse editing method. (f) Rapid erasing enabled by the dual‐pulse editing method. (g) Retention characteristics of the multilevel states. (h) Endurance performance under repeated programming/erasing operation.

A related approach was demonstrated by Cai et al. [119], who introduced a coplanar control‐gate geometry to engineer the capacitive coupling coefficient between the gate and the channel (Figure 18a). By tuning the area ratio and using a dual‐pulse programming scheme, the device achieved 256 clearly distinguishable conductance states (8‐bit precision), with neighboring states separated by more than 3σ (Figure 18b). These states remained stable for over 10 000 s (Figure 18c) and survived more than 10^5^ programming/erasing cycles (Figure 18d). Beyond device‐level characterization, an experimentally fabricated 9 × 2 array (Figure 18e) was used for image convolution and vector‐matrix multiplication (Figure 18f), and the experimentally programmed conductance distribution closely matched the trained software weights (Figure 18g). In an ImageNet recognition task, the system maintained near‐ideal inference accuracy even under 8‐bit quantization (Figure 18h,i). These results demonstrate that multilevel storage in 2D flash memory is not merely a memory feature, but a practical enabler of high‐precision analog computing.

**FIGURE 18:**
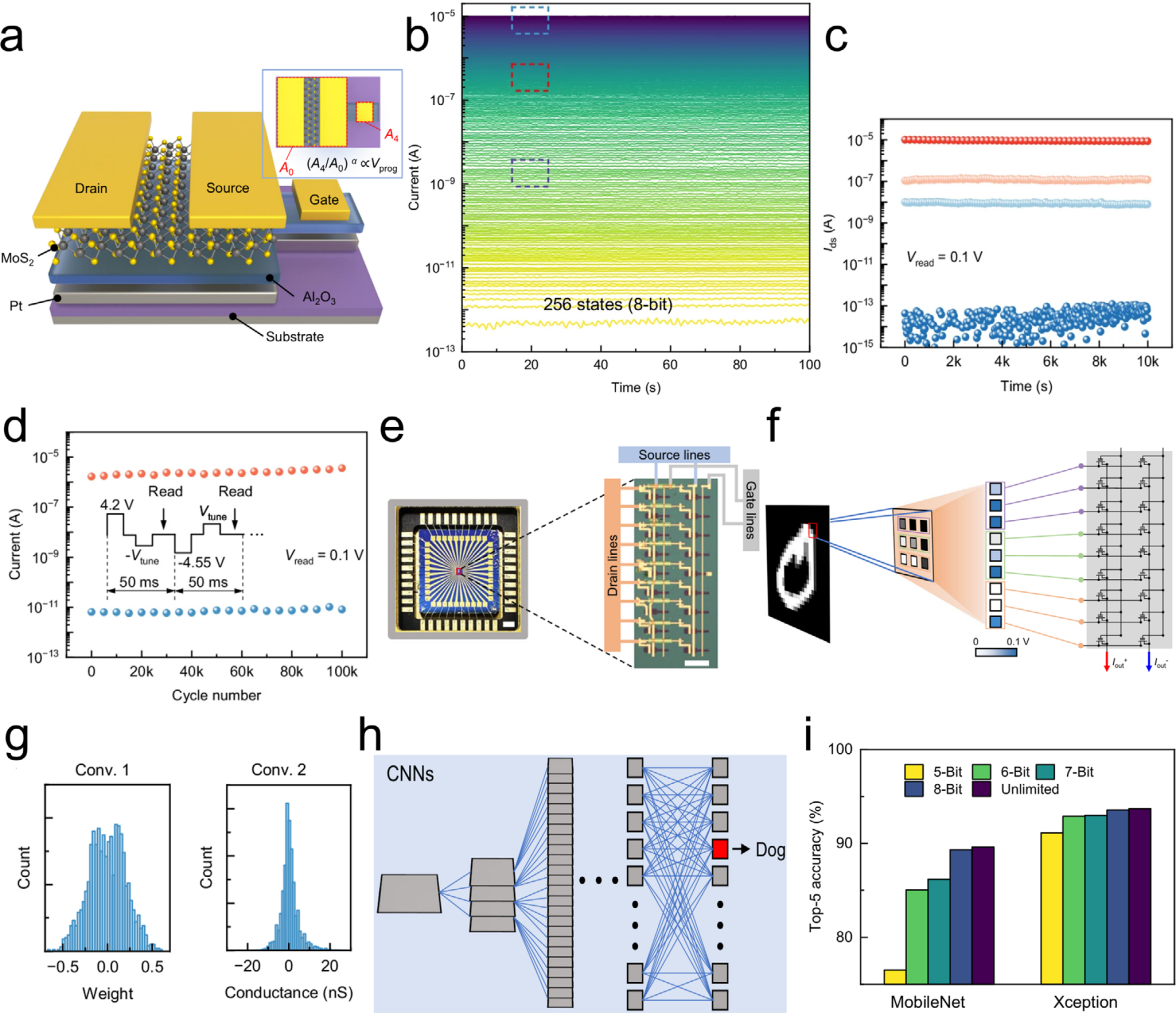
Low‐voltage, high‐precision multilevel storage and array‐level computing demonstration [119]. (a) Schematic of the gate‐injection 2D floating‐gate memory with coplanar gate geometry. (b) Time‐dependent current characteristics of 8‐bit memory states. (c) Retention performance of the programmed multilevel states. (d) Endurance performance during repeated programming/erasing cycles. (e) Schematic of the 9 × 2 memory array. (f) Vector‐matrix multiplication scheme for image convolution. (g) Comparison between hardware‐programmed conductance distribution and software‐trained weights. (h) Convolutional neural‐network architecture for ImageNet recognition. (i) Inference accuracy under different quantization precisions.

For analog in‐memory computing, the number of distinguishable conductance states mainly reflects the resolution of weight storage, whereas the actual weight‐update characteristics should also be evaluated in terms of the linearity and symmetry of conductance modulation [120]. In flash‐memory devices, as stored charge accumulates or is released, the floating‐gate potential and the local electric field in the tunneling region change accordingly, making subsequent charge injection and release processes state dependent. Therefore, the number of distinguishable conductance states alone cannot fully describe the weight‐update behavior. Accordingly, when evaluating 2D flash memory for analog in‐memory computing, additional factors such as dynamic range, update linearity, symmetry between potentiation and depression, device‐to‐device uniformity, and long‐term stability should also be considered [121].

### 4.2 Integrated Sensing‐Memory‐Computing

Beyond multilevel storage, the next step is the integration of sensing, memory, linear computation, and nonlinear activation within a unified hardware platform. Such sensing‐memory‐computing architectures aim to eliminate repeated data transfer among sensors, memory arrays, processors, and activation circuits, thereby further improving parallelism and energy efficiency [122, 123, 124].

Zhang et al. [36] proposed a reconfigurable multifunctional split‐floating‐gate memory (MM‐SFGM) capable of performing in‐sensor computing, in‐memory computing, and nonlinear activation functions within the same device (Figure 19a). The structure employs spatially separated dual gates, enabling independent control over carrier type and local potential. As a result, the same channel can be reconfigured into *p‐n, n‐p, n‐n*, and *p‐p* homojunctions (Figure 19b).

**FIGURE 19:**
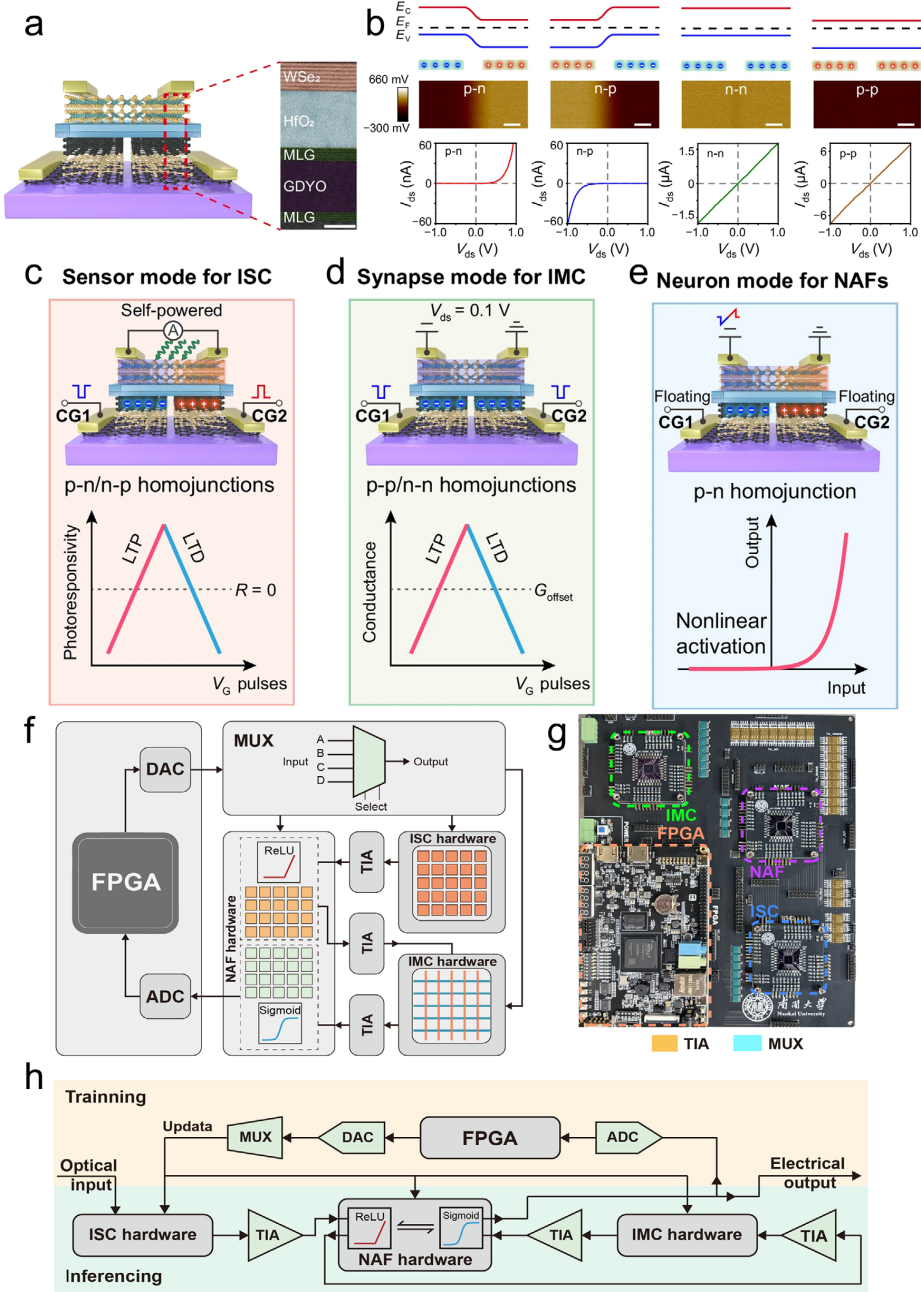
Multifunctional split‐floating‐gate memory for integrated sensing‐memory‐computing [36]. Reproduced under terms of the CC BY 4.0 license [36]. Copyright 2026, Springer Nature. (a) Schematic of the multifunctional split‐floating‐gate memory device. (b) Reconfigurable *p‐n, n‐p, n‐n*, and *p‐p* homojunction configurations and corresponding transfer characteristics. (c) Operation in the in‐sensor‐computing mode, where photoresponse and nonvolatile memory are coupled. (d) Operation in the in‐memory‐computing mode, where analog conductance states support weight storage and update. (e) Nonlinear activation functions enabled by reconfigurable rectification behavior. (f) Schematic of the complete hardware architecture integrating sensing, memory, computing, and activation. (g) Photograph of the assembled hardware prototype. (h) Operational flow of the integrated sensing‐memory‐computing system.

In the *p‐n* and *n‐p* configurations, the device acts as a self‐powered photodetector (Figure 19c). In this mode, the photoresponsivity can be linearly tuned and nonvolatilely stored, while 63 distinguishable analog states are obtained with extremely low programming energy. Because the photocurrent scales linearly with light intensity, multiply‐accumulate operations can be directly performed at the sensor level. In the *p‐p* and *n‐n* configurations, the device operates in a memory‐computing mode (Figure 19d), in which conductance can be linearly and symmetrically updated over a broad range, again supporting 63 stable analog states and endurance over 10^6^ cycles.

By tuning the floating‐gate charge state, the rectification behavior of the same device can be reconfigured to realize ReLU‐like and Sigmoid‐like nonlinear activation responses (Figure 19e). In this way, both weight storage and activation are integrated at the device level, eliminating one of the most persistent separations in hardware neural networks. At the system level, the MM‐SFGM array was integrated into a complete hardware framework incorporating sensing, memory, linear computing, and nonlinear activation modules, and was experimentally validated on a prototype platform with FPGA‐assisted control (Figure 19f–h). This work demonstrates that 2D flash memory can evolve beyond storage and in‐memory computing toward a fully integrated sensing‐memory‐computing‐activation paradigm.

### 4.3 From Device Arrays to Practical Systems

While single‐device demonstrations establish the fundamental feasibility of 2D flash memory, their practical impact ultimately depends on scalability toward arrays and system‐level implementations [125]. In this context, the performance of 2D flash memory should not be evaluated solely in terms of device‐level metrics such as the number of conductance states or energy per operation. More importantly, array‐level characteristics—including device‐to‐device variability, programming linearity, and noise accumulation during repeated updates—play a decisive role in determining computational accuracy and system reliability [126].

The multilevel and multifunctional examples discussed above highlight two key system‐level principles. First, precise analog‐state programmability must be accompanied by low variability and noise to preserve fidelity across large‐scale arrays. Second, the functional advantage of 2D flash memory becomes significantly more pronounced when a single physical platform supports not only storage, but also sensing, reconfigurable transport, and nonlinear response.

These considerations indicate that the future development of 2D flash memory extends beyond optimizing individual device performance toward achieving robust, uniform, and computation‐ready array architectures [127, 128]. In this sense, 2D flash memory is emerging as a multifunctional hardware substrate capable of supporting scalable, data‐centric computing systems beyond the conventional von Neumann paradigm.

As summarized in Figure 20, these properties collectively drive the evolution of 2D flash memory from single‐device demonstrations toward large‐scale array integration, application‐specific computing systems, and CMOS‐compatible chip‐level implementations. However, this transition also involves several key challenges, including device‐to‐device variability and array uniformity, crosstalk and program/read disturbance at the array level, as well as thermal‐budget and process‐compatibility constraints for CMOS back‐end integration. Consequently, advancing 2D flash memory toward practical systems will require more systematic co‐optimization of device uniformity, array scalability, and CMOS integration compatibility.

**FIGURE 20:**
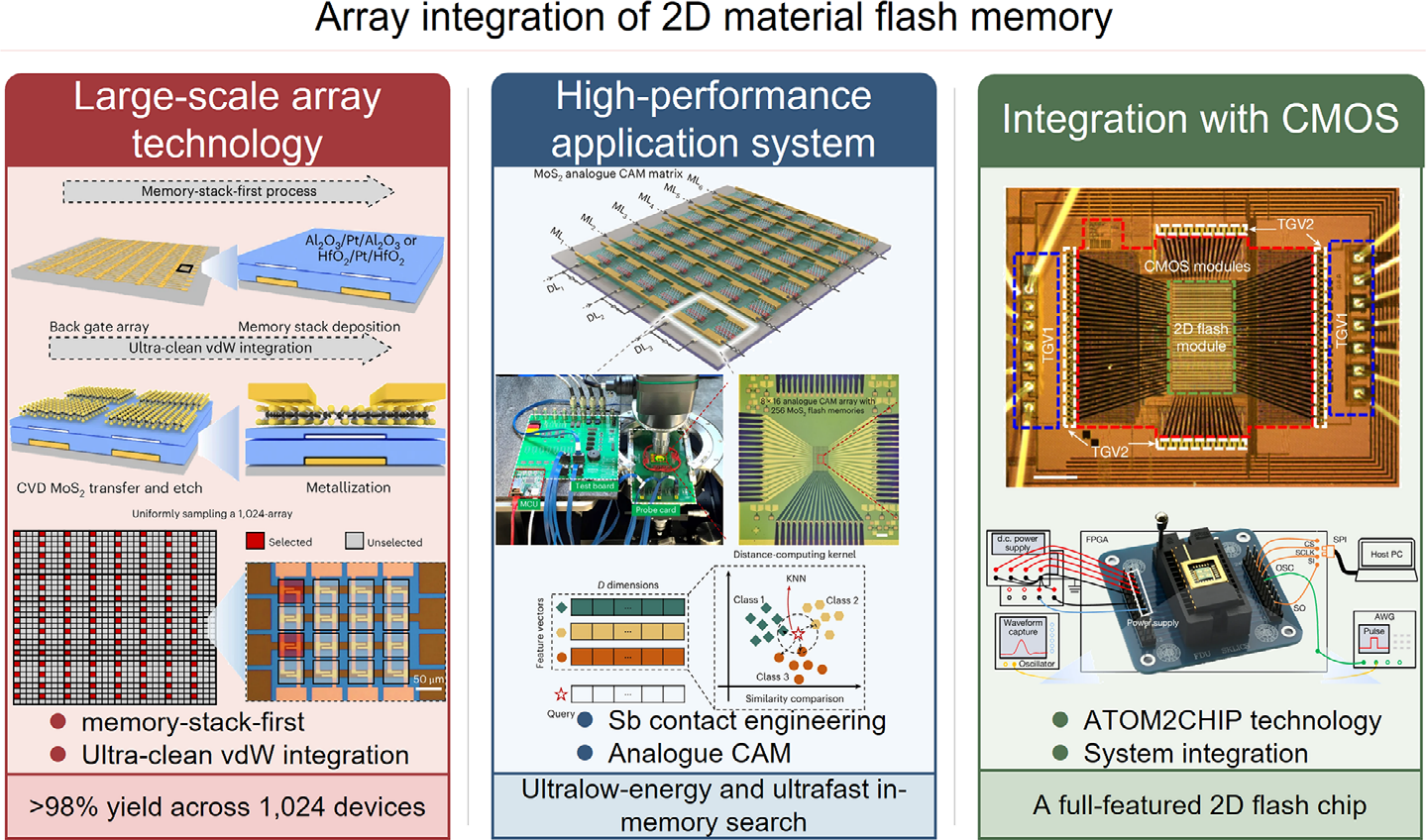
Evolution of 2D flash memory from single devices to practical systems [37, 129, 130]. Schematic roadmap showing the transition of 2D flash memory from individual device demonstrations to large‐scale arrays, application‐oriented computing systems, and CMOS‐compatible chip‐level integration. The evolution involves improvements in array uniformity, high‐density integration, content‐addressable search, in‐sensor computing, and heterogeneous system‐on‐chip implementation.

#### 4.3.1 Large‐Scale Array Fabrication

To achieve large‐scale array integration, Jiang et al. [129] proposed an ultra‐clean and scalable van der Waals integration strategy based on a memory‐stack‐first process (Figure 21a), which effectively minimizes interfacial contamination and process‐induced damage, thereby ensuring high interface quality and device uniformity. The devices exhibit ultrafast programming at approximately 20 ns (Figure 21b). The process is also compatible with multiple storage stacks, including Al_2_O_3_/Pt/Al_2_O_3_. Using monolayer MoS_2_ and the HfO_2_/Pt/HfO_2_ stack (Figure 21c), the authors experimentally fabricated a 1024‐cell flash array with high electrical uniformity and a yield of 98.4% (Figure 21d).

**FIGURE 21:**
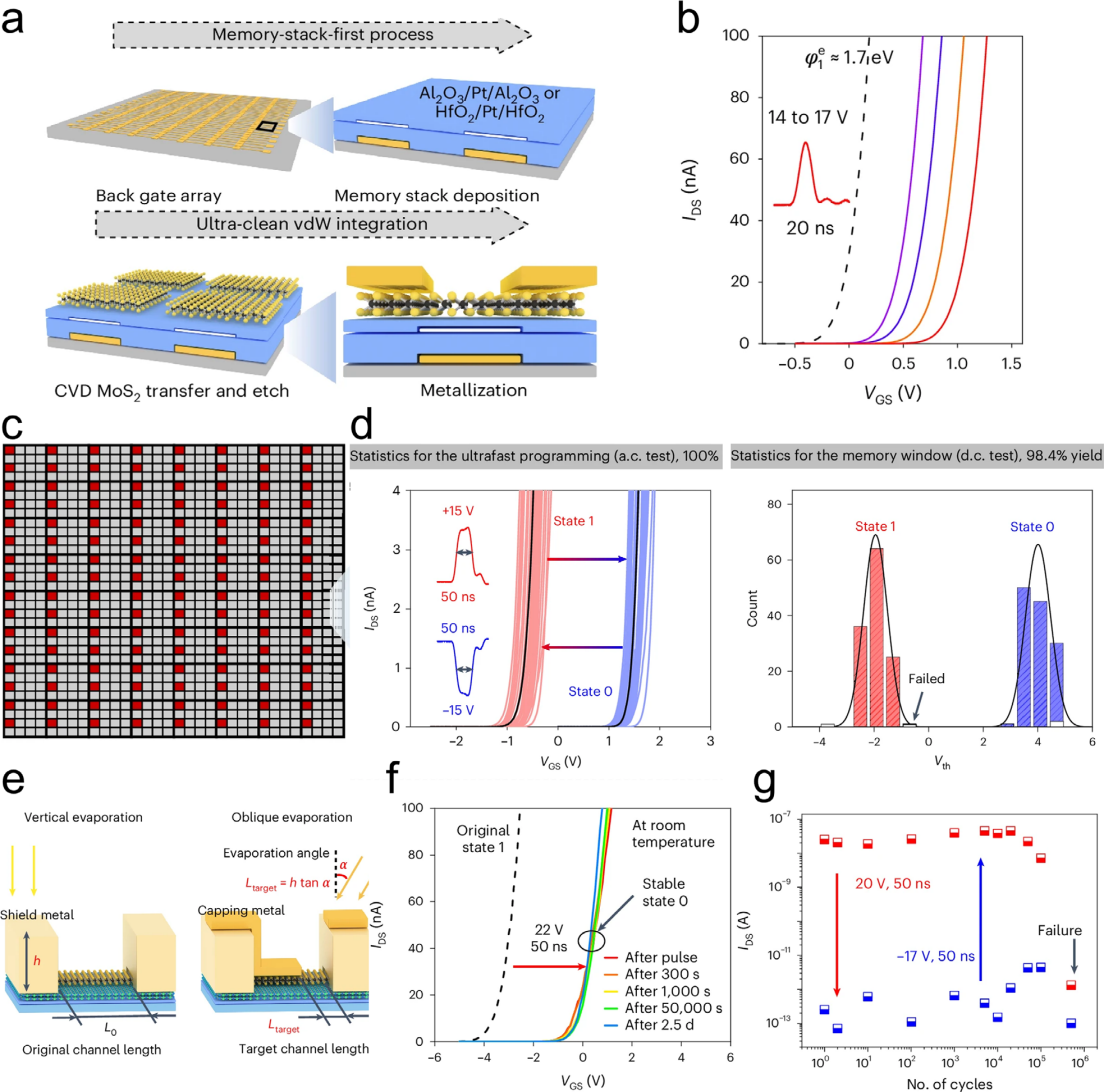
Scalable fabrication of large‐area 2D flash arrays via ultraclean van der Waals integration [129]. Reproduced with permission [129]. Copyright 2024, Springer Nature. (a) Schematic of the memory‐stack‐first process for scalable array fabrication. (b) Ultrafast programming characteristics of the HfO_2_/Pt/HfO_2_‐based flash memory. (c) Integrated 1024‐cell 2D flash‐memory array. (d) Statistical distributions of programming performance and device yield. (e) Schematic of sub‐10 nm channel fabrication by shadow evaporation. (f) Retention performance of the sub‐10 nm devices. (g) Endurance performance of the sub‐10 nm devices.

To further improve integration density, a composite Al_2_O_3_/HfO_2_/hBN storage stack combined with oblique‐angle evaporation is employed to fabricate sub‐10 nm channel devices (Figure 21e). This architecture suppresses short‐channel effects while maintaining electrostatic control, enabling aggressive scaling. Even at such reduced dimensions, the devices maintain stable threshold voltages and reliable switching behavior during long‐term retention (Figure 21f) and repeated programming/erasing cycles (Figure 21g). These results demonstrate that scalable fabrication, high‐yield integration, and extreme device scaling can be simultaneously achieved through careful co‐design of materials and processes.

#### 4.3.2 High‐Performance Application Systems

Building upon array‐level integration, Gao et al. [130] developed an analogue content‐addressable memory (CAM) system based on MoS_2_ flash devices with Sb/Au contacts (Figure 22a). Unlike conventional metal contacts, the semimetallic Sb contact suppresses metal‐induced gap states and reduces Fermi‐level pinning, enabling low‐resistance charge injection (Figure 22b). As a result, the device exhibits highly linear output characteristics and enhanced readout current up to 60 µA µm^−^^1^ and an on/off ratio exceeding 10^9^ (Figure 22c).

**FIGURE 22:**
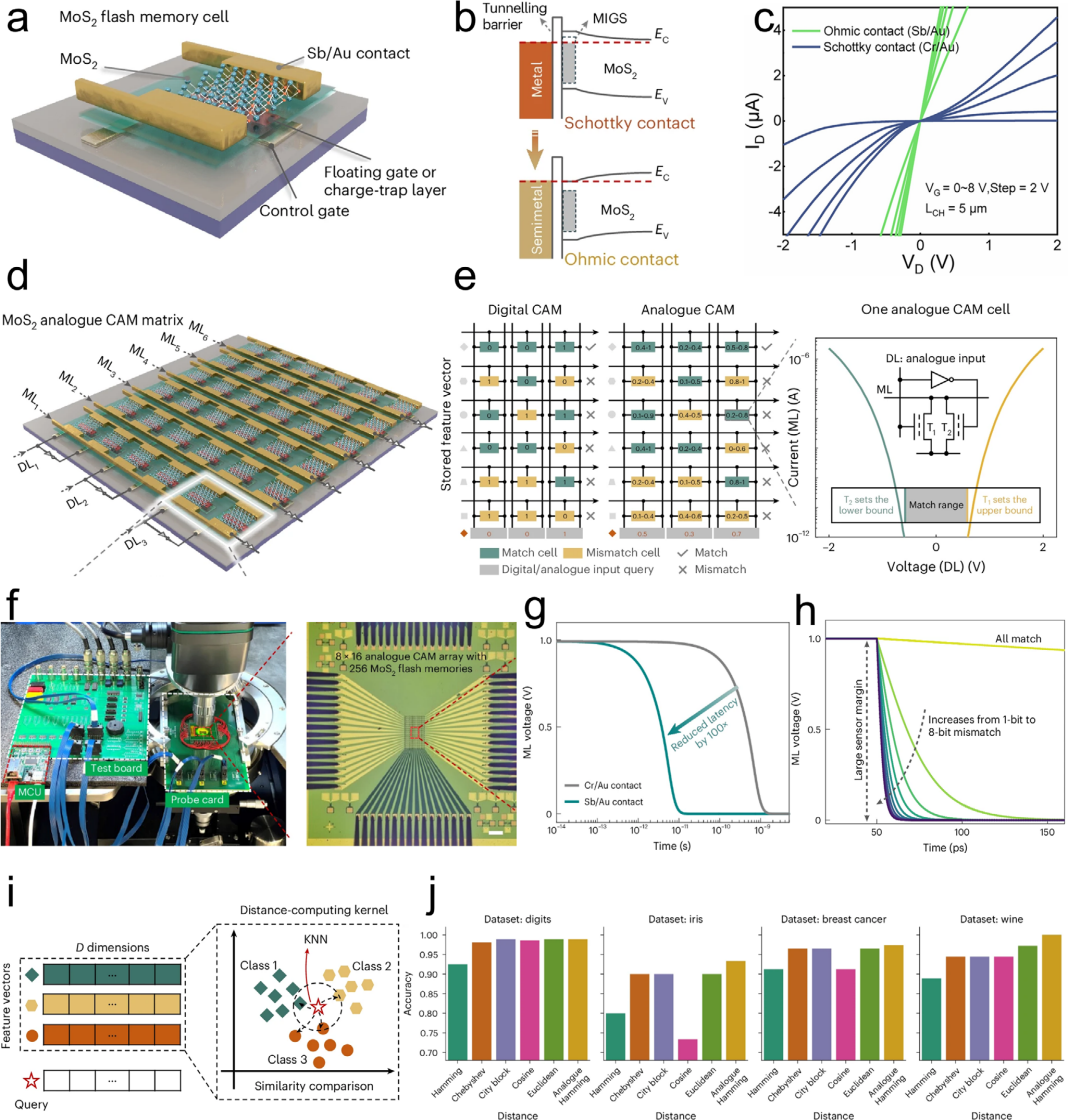
Array‐level analogue content‐addressable memory system based on MoS_2_ flash memory [130]. Reproduced with permission [130]. Copyright 2025, Springer Nature. (a) Schematic of the MoS_2_ flash memory device with semimetal‐assisted contact engineering. (b) Energy‐band diagrams comparing Schottky and ohmic contacts at the metal/MoS_2_ interface. (c) Output characteristics of semimetal‐contacted and conventional‐contacted devices. (d) Schematic of the analogue content‐addressable memory array. (e) Operating principle of continuous‐range analogue matching. (f) Schematic and optical image of the fabricated analogue CAM hardware system. (g) Reduced search latency enabled by high‐current and low‐leakage MoS_2_ flash cells. (h) Simulated discharge behavior for all‐match and multi‐bit mismatch cases. (i) Analogue CAM‐based KNN classification scheme. (j) Classification accuracy across different datasets and distance metrics.

An 8 × 16 analogue CAM array is constructed (Figure 22d), where programmable threshold‐voltage tuning enables continuous‐range matching (Figure 22e). A dedicated hardware platform is further implemented to validate array‐level operation (Figure 22f). Benefiting from the high readout current and low leakage enabled by the Sb/Au contacts, the system shows markedly reduced search latency (Figure 22g) and can clearly distinguish all‐match from multi‐bit mismatch cases in simulation (Figure 22h). The system is estimated to achieve an energy consumption below 0.1 fJ per search per cell and an ultralow latency of 36 ps, representing a 13.8× improvement in energy efficiency and a 568× reduction in latency compared with conventional SRAM‐based CAM systems.

At the application level, the system further enables similarity search and high‐accuracy KNN classification across multiple datasets (Figure 22i,j). These results highlight the potential of 2D flash‐based analogue CAM systems for energy‐efficient and high‐speed in‐memory computing applications.

#### 4.3.3 Integration with CMOS

Beyond array and system demonstrations, practical deployment of 2D flash memory requires seamless integration with CMOS platforms. In view of the incompatibility between 2D materials and CMOS processes [131, 132]. Liu et al. [37] addressed this using an atom‐to‐chip (ATOM2CHIP) strategy, enabling monolithic integration of 2D flash modules on CMOS back‐end metal layers (Figure 23a). A modular 3D architecture separates the 2D memory core from CMOS circuits and reconnects them through dedicated interfaces (Figure 23b), reducing system integration to interface engineering.

**FIGURE 23:**
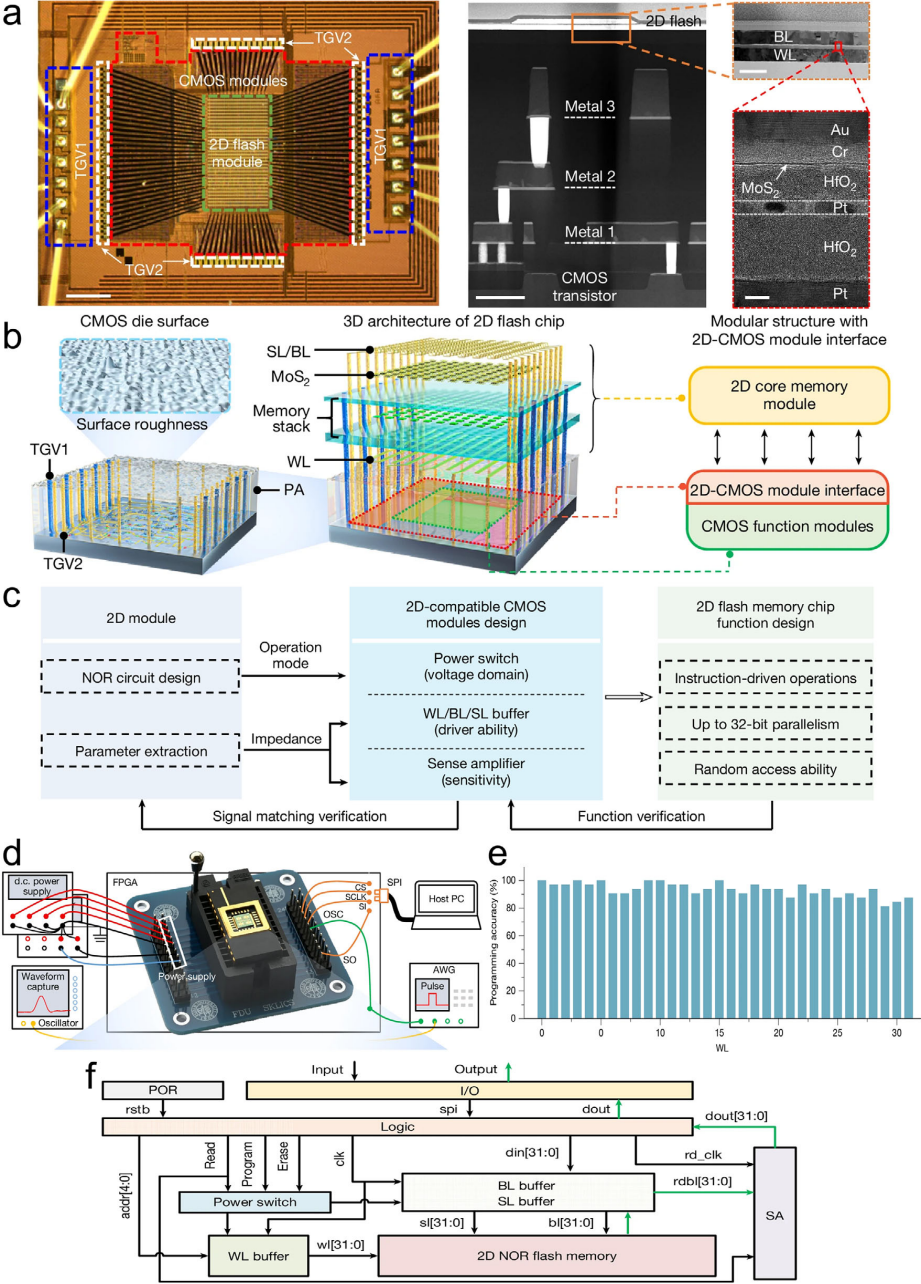
Heterogeneous integration of 2D flash memory with CMOS platforms [37]. Reproduced with permission [37]. Copyright 2025, Springer Nature. (a) Optical image and cross‐sectional TEM images of the monolithically integrated 2D flash‐CMOS chip. (b) 3D architecture of the fabricated 2D flash chip, showing the separation and interconnection of the 2D memory module and CMOS circuitry. (c) Cross‐platform compatibility verification and co‐design methodology for 2D‐material/CMOS integration. (d) Schematic of the chip test system. (e) Data flow inside the 2D flash chip during instruction‐driven operation. (f) Programming accuracy across 32 word lines after checkerboard programming, demonstrating chip‐level functionality and integration uniformity.

Through conformal integration, 2D‐compatible packaging, and cross‐platform co‐design (Figure 23c), electrical matching between memory and CMOS circuits is achieved. The chip exhibits a yield of 94.34%, an operation speed of ∼20 ns, and an energy consumption as low as 0.644 pJ per bit. FPGA‐based testing confirms instruction‐driven operation, 32‐bit parallel programming, and random access (Figure 23d,e), with high programming accuracy across 32 word lines (Figure 23f). This work establishes a viable route toward fully integrated 2D flash systems. By bridging the gap between device demonstrations and chip‐scale integration, it provides a critical foundation for positioning 2D flash memory as a scalable and computation‐ready hardware platform for next‐generation data‐centric systems.

## 5 Conclusions and Outlook

### 5.1 Design Principles for High‐Quality 2D Flash Memory

For a next‐generation flash memory technology to become practically competitive, high programming speed, low operating energy, long retention, strong endurance, and scalable integration must be achieved simultaneously. The representative 2D flash memory devices discussed in this Review demonstrate that these metrics are not determined by a single material parameter or device component. Instead, they emerge from the coordinated regulation of charge injection, transport, trapping, and confinement across atomically thin channels, tunneling barriers, charge‐storage layers, and van der Waals interfaces. Table 2 summarizes the performance of recently reported 2D flash memory devices and reveals a central message: the advantages of 2D flash memory arise from a transition from bulk‐field‐driven charge programming to interface‐controlled charge programming.

**TABLE 2:** Performance comparison of representative 2D flash memory devices.

Structure	Programming condition	Speed	Voltage	Energy	Retention	Endurance (cycles)	Key feature	Refs.
MoS_2_/hBN/Te	500 ns / −12 V; 100 ns / 12 V	ns	∼12 V	—	>4 × 10^3^ s	>4 × 10^3^	van der Waals heterostructure memory	[133]
PtS_2_/hBN/MLG	40 ns / −5.5 V; 40 ns / 5.5 V	ns	∼5.5 V	—	>10^4^ s	>10^5^	Low‐voltage van der Waals device	[83]
MoS_2_/hBN/FLG	10 ns / −7 V; 100 ns / 15 V	ns	7–15 V	—	>10^4^ s	>10^6^	Edge‐contact enhanced injection	[34]
Graphene/hBN/HfO_2_/Al_2_O_3_	400 ps / −5 V	sub‐ns	∼5 V	—	>6 × 10^4^ s	>5 × 10^6^	2D‐enhanced hot‐carrier injection	[45]
MoS_2_/hBN/GDY/Gr	8 ns / 30 mV; 100 ns / 30 mV	ns	30 mV	—	>10^4^ s	10^3^	Ultralow‐voltage programming	[100]
MoS_2_/hBN/MoS_2_/GDYO/WSe_2_	20 ns / ± 2 V	ns	∼2 V	10 fJ	>10^4^ s	10^3^	Threshold‐switching‐assisted injection	[101]
WS_2_/hBN/WS_2_	1.5 s / −10 V; 1.5 s / 2 V	s	∼10 V	—	10^4^ s	10^3^	Gate‐tunable contact modulation	[102]
MoS_2_/PbI_2_	5 s / 40 V	s	40 V	—	>1.2 × 10^4^ s	>10^4^	Intrinsic deep‐level traps	[103]
MoS_2_/CdSe@CdS QDs	1 s / 80 V	s	80 V	—	5 × 10^4^ s	>5 × 10^4^	Quantum‐dot trapping layer	[104]
MoS_2_/Al_2_O_3_/Pt	90 ns; 230 ns	ns	—	—	>10^4^ s	>10^5^	Gate‐injection architecture	[35]
MoS_2_/Al_2_O_3_/Pt	5 s / ∼3 V	s	∼3 V	—	10^4^ s	10^5^	Low‐voltage FG memory	[119]
WSe_2_/HfO_2_/MLG/GDYO/MLG	20 ns / ±1.2 V	ns	∼1.2 V	4.8 fJ	>10^5^ s	>10^6^	Ultra‐low‐power multilevel memory	[36]
MoTe_2_/hBN/graphene	1 s / 30 V	s	30 V	—	>3 × 10^3^ s	>5 × 10^2^	van der Waals floating‐gate device	[134]
InSe/hBN/MLG	21 ns / ± 20 V	ns	∼20 V	—	16.5 days	2 × 10^3^	High‐quality van der Waals interface	[99]
MoS_2_/hBN/MLG	20 ns / 25–30 V	ns	25‐30 V	—	5300 s	1390	Conventional van der Waals FG memory	[135]
MoS_2_/hBN/MLG/hBN/MLG	20 ns / ∼29 V	ns	∼29 V	—	10^5^ s	4200	Multi‐layer storage structure	[136]
MoS_2_/TaO_x_/TaS_2_	1 s / 10 V	s	10 V	—	>10^4^ s	10^3^	Oxide‐assisted trapping	[137]
ReS_2_/hBN/graphene	1 s / 20 V	s	20 V	—	4000 s	400	van der Waals memory device	[138]
WSe_2_ (oxidized)	100 ms / ± 6 V	ms	∼6 V	—	10^4^ s	>5 × 10^3^	Defect‐engineered channel	[139]
hBN/SLG/hBN	100 ms / ± 20 V	ms	20 V	—	>1 year	9800	All‐2D floating‐gate memory	[140]

As summarized in Table 2, the performance of representative 2D flash memory devices spans a broad range of programming speeds, operating voltages, and reliability metrics, reflecting the diversity of material systems and structural designs. Notably, ultrafast programming down to the subnanosecond regime has been achieved through engineered injection pathways such as 2D‐enhanced hot‐carrier injection and direct tunneling, while low‐voltage operation below a few volts is enabled by optimized band alignment and reduced tunneling barriers. In parallel, deep trapping layers and high‐quality van der Waals interfaces contribute to improved retention and endurance. Importantly, these results reveal that the performance enhancement in 2D flash memory does not originate from a single design parameter, but from the coordinated control of charge injection, transport, trapping, and confinement. This unified interface‐controlled framework provides a viable pathway to partially decouple the conventional trade‐off among speed, power consumption, and reliability, which remains a fundamental limitation in silicon‐based flash memory.

From the perspective of programming speed, 2D flash memory has progressed from microsecond‐ or millisecond‐scale operation toward nanosecond, subnanosecond, and even picosecond regimes. This acceleration is mainly enabled by reducing the effective injection barrier and shortening the charge‐transfer pathway through phase‐engineered contacts, van der Waals tunneling layers, direct tunneling, and 2D‐enhanced hot‐carrier injection. Unlike conventional flash memory, where faster programming generally requires stronger electric fields and therefore accelerates dielectric degradation, 2D flash devices can redistribute the programming field across engineered interfaces and atomically thin channels. This offers a route to increase injection efficiency without proportionally increasing electrical stress.

For low‐power operation, the key requirement is not only to reduce the programming voltage, but also to suppress programming current and shorten the switching time. Recent 2D flash devices have demonstrated low‐voltage and femtojoule‐level operation by combining favorable band alignment, high‐quality tunneling barriers, threshold‐switching‐assisted injection, and gate‐tunable contact modulation. These results indicate that energy consumption in 2D flash memory can be minimized through three coupled strategies: lowering the injection barrier, improving electrostatic gate control, and reducing leakage pathways during both programming and retention.

Retention and endurance remain the most stringent reliability metrics for practical flash technologies. Long retention requires deep and stable trapping states, suppressed back tunneling, and minimized leakage through the tunneling dielectric and surrounding interfaces. High endurance further requires that charge injection and removal occur without severe defect generation, trap evolution, or irreversible interfacial degradation. In this regard, van der Waals heterostructures provide a unique advantage because they can form atomically sharp and dangling‐bond‐free interfaces, while 2D storage media, quantum dots, ferroelectric layers, and defect‐engineered trapping layers provide additional freedom for stabilizing stored charges [141]. However, the retention and endurance of many reported 2D flash devices are still evaluated under nonstandard conditions, making rigorous benchmarking essential for future progress.

Several general design principles can therefore be extracted. First, contact engineering is central to high‐performance 2D flash memory because charge injection in atomically thin channels is highly sensitive to contact barriers, Fermi‐level pinning, and local electric‐field distribution. Phase‐engineered edge contacts, semimetal contacts, and gate‐tunable Schottky/quasi‐ohmic contacts can all substantially improve injection efficiency. Second, the tunneling layer must be optimized as the core element governing the speed‐retention trade‐off. It should be thin enough to permit fast charge transfer but sufficiently uniform and robust to suppress leakage and cycling‐induced degradation. Third, the storage layer should provide a high density of stable and energetically suitable trapping sites, rather than simply maximizing trap density. Fourth, the full heterostructure must be designed at the band‐alignment level so that charge injection, trapping, confinement, and release are all controlled in a coordinated manner.

Taken together, 2D flash memory should not be evaluated only by isolated record values in speed, voltage, retention, or endurance. Its broader significance lies in the possibility of designing the full lifecycle of stored charges through interfaces. Efficient injection can be achieved through contact and barrier engineering; low‐energy operation can be realized through band‐structure and dielectric design; long‐term retention can be improved through trap‐state and leakage‐pathway control; and high endurance can be obtained by reducing electrical and structural stress during repeated cycling. This interface‐controlled framework provides the physical basis for mitigating the conventional trade‐offs among speed, power consumption, and reliability.

### 5.2 Outlook

Overall, 2D flash memory is transitioning from single‐device performance demonstrations toward functionally enriched and system‐oriented applications. To date, most efforts have focused on improving programming speed, reducing operating voltage and energy consumption, enhancing retention and endurance, and increasing scalability through material selection, interface optimization, and heterostructure design. However, the true significance of 2D flash memory may go far beyond that of a high‐performance replacement for existing nonvolatile memories. It offers a pathway to break the conventional separation among logic, memory, and computation, thereby enabling hardware platforms in which different functions are structurally unified rather than externally interconnected.

One particularly important future direction lies in redefining of hysteresis. In logic transistors, hysteresis is often treated as an undesirable parasitic effect because it introduces history dependence and uncertainty [142, 143, 144, 145]. In nonvolatile memory, by contrast, hysteresis is precisely the physical basis of writing and retention [146, 147]. Reconciling these apparently contradictory requirements within a single device platform will be a central scientific challenge for future reconfigurable and multifunctional electronics [148]. Thus, future studies should not only pursue larger memory windows, faster programming, or longer retention, but should also explore the on‐demand activation, precise regulation, and functional use of hysteresis itself, turning it from a passive by‐product into a programmable resource. Controllable hysteresis can be achieved by engineering interface traps, charge migration, or ferroelectric polarization [149]. Reconfigurable hysteresis provides a new design strategy for circuit‐level functional reconfiguration, enabling the same device to switch between memory and logic‐transistor operating modes, thereby providing a basis for in‐memory computing and monolithic logic–memory integration [150]. In the future, controllable hysteresis may be further extended toward coordinated operation among multiple devices, while the stable and reproducible utilization of different hysteresis states at the circuit level should also be explored. These advances could promote its application in reconfigurable logic, in‐memory computing, and integrated logic–memory circuits.

The functional landscape of 2D flash memory is expanding rapidly. Through fine control of injected charge, multilayer or split‐floating‐gate architectures, and coupling with optical, electrical, or other external stimuli, 2D flash devices are evolving into integrated systems that combine sensing, storage, computation, and activation [151, 152]. In this sense, 2D flash memory is an emerging hardware platform capable of unifying information storage, state programmability, and in situ computing for the post‐Moore era.

Nevertheless, substantial challenges remain before 2D flash memory can move from the laboratory to practical deployment. First, many record‐level performances are still based on single devices or small‐scale arrays and remain far from the levels of uniformity, yield, and robustness required for large‐scale manufacturing. Wafer‐scale synthesis, thickness control, defect engineering, and cross‐wafer uniformity of 2D materials all remain challenging, while transfer‐related contamination, wrinkles, cracks, and residual interfacial impurities further amplify variability. Second, when devices are scaled into arrays, issues such as crosstalk, leakage, voltage nonuniformity, program/read disturbance, and compatibility with peripheral circuitry must be systematically addressed. These problems become even more severe in multilevel and analog storage modes, where state drift, noise accumulation, and device variability can significantly affect array‐level precision and system reliability. In addition, if 2D flash memory is to be deeply integrated with existing CMOS platforms, it must satisfy the thermal budget of back‐end integration and achieve further progress in materials compatibility, patterning, interconnect formation, and BEOL process integration.

Ultimately, the practical adoption of 2D flash memory will not be determined by record performance in any single device metric, but by whether co‐optimization across materials, interfaces, mechanisms, fabrication, and system architecture can be achieved. With continued advances in interface engineering, field‐coupled modulation, large‐area integration, and cross‐layer hardware design, 2D flash memory is poised to become not merely a supplement to current memory technologies, but a foundational device platform for reconfigurable electronics, in‐memory computing architectures, and intelligent sensing hardware.

## Author Contributions

**Zhiyuan Wang**: conceptualization, writing – original draft, formal analysis, Writing – review and editing, investigation. **Fanyi Meng**: writing – original draft, writing – review and editing, funding acquisition, project administration, supervision. **Fanying Meng**: writing – original draft. **Shida Huo**: writing – original draft. **Junxi Xu**: writing – original draft. **Yaohui Wang**: writing – original draft. **Yuexuan Ma**: writing – original draft. **Yuan Xie**: writing – original draft, writing – review and editing, formal analysis. **Xiaodong Hu**: writing – original draft. **Caofeng Pan**: writing – original draft, writing – review and editing, supervision. **Enxiu Wu**: conceptualization, writing – original draft, writing – review and editing, project administration, supervision, resources.

## Conflicts of Interest

The authors declare no conflicts of interest.

## Data Availability

The data that support the findings of this study are available in the supplementary material of this article.
